# Cilia‐Mimic Locomotion of Magnetic Colloidal Collectives Enhanced by Low‐Intensity Ultrasound for Thrombolytic Drug Penetration

**DOI:** 10.1002/advs.202410351

**Published:** 2024-12-27

**Authors:** Jingjing Wu, Weijuan Zou, Qijie Lu, Tingjia Zheng, Yanping Li, Tao Ying, Yuehua Li, Yuanyi Zheng, Longchen Wang

**Affiliations:** ^1^ Department of Ultrasound in Medicine Shanghai Sixth People's Hospital Affiliated to Shanghai Jiao Tong University School of Medicine No. 600, Yishan Road Shanghai 200233 P. R. China; ^2^ Shanghai Key Laboratory of Neuro‐Ultrasound for Diagnosis and Treatment Shanghai 200233 P. R. China; ^3^ Department of Radiology Shanghai Sixth People's Hospital Affiliated to Shanghai Jiao Tong University School of Medicine No. 600, Yishan Road Shanghai 200233 P. R. China

**Keywords:** ciliary locomotion, drug penetration, low‐intensity ultrasound, magnetic nanorobot, thrombolysis

## Abstract

Rapid thrombolysis is very important to reduce complications caused by vascular blockage. A promising approach for improving thrombolysis efficiency is utilizing the permanent magnetically actuated locomotion of nanorobots. However, the thrombolytic drug transportation efficiency is challenged by in‐plane rotating locomotion and the insufficient drug penetration limits further improvement of thrombolysis. Inspired by ciliary movement for cargo transportation in human body, in this study, cilia‐mimic locomotion of magnetic colloidal collectives is realized under torque‐force vortex magnetic field (TFV‐MF) by a designed rotating permanent magnet assembly. This cilia‐mimic locomotion mode can generate more disturbances to the fluids to improve thrombolytic drug transportation and the increased height and area of colloidal collectives boosted the imaging capability. In addition, low‐intensity ultrasound is applied to enhance colloids infiltration by producing the fiber breakage and inducing erythrocyte deformation. In vitro thrombolytic experiments demonstrate that the thrombolysis efficiency increased by 16.2 times compared with that of pure tissue plasminogen activator (tPA) treatments. Furthermore, in vivo rat models of femoral vein thrombosis confirmed that this approach can achieve blood flow recanalization more quickly. The proposed cilia‐mimic locomotion of magnetic colloidal collectives combined with low‐intensity ultrasound irradiation mode provides a new insight of therapeutic interventions for vascular thrombus by enhancing drug penetration.

## Graphical abstract

This study develops an approach to improve the penetration of thrombolytic drugs by the regulating cilia‐mimic locomotion of colloidal collectives under a torque‐force vortex magnetic field with low‐intensity ultrasound irradiation, providing a new insight of therapeutic interventions for vascular thrombus.

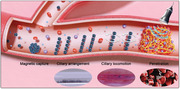

## 1 Introduction

Thrombi can cause many life‐threatening conditions, such as ischemic stroke and pulmonary embolism.^[^ ^1^, ^2^ ^]^ The main clinical methods used to treat thrombosis are interventional surgery and drug therapy; however, interventional thrombectomy involves several disadvantages. For example, physicians must possess a high level of technical expertise, patients are exposed to radiation from X‐rays, the narrow vasculature cannot be easily accessed by catheters, and the procedure involves surgical risks.^[^ ^3^ ^]^ In contrast, intravenous thrombolytic drug treatment with recombinant human tissue plasminogen activator (tPA), which is a clinical commonly used drug approved by Food and Drug Administration (FDA), is convenient and feasible. However, the efficiency of thrombolysis is limited by the concentration of drugs that reach the thrombus site, and increasing the dosage of injected drugs may lead to various risks, such as intracranial hemorrhage.^[^ ^4^, ^5^ ^]^ Additionally, the time window for drug treatment is only a few hours.^[^ ^6^ ^]^ Therefore, improving thrombolysis efficiency is crucial for expanding the application of intravenous thrombolytic drug treatment.

As nanotechnology and automatic control technology has advanced, micro/nano‐robots have shown good clinical application prospects in delivering drugs.^[^ ^7^ ^]^ Among the various driving methods, magnetic actuated micro/nanorobots have attracted the interest of many researchers.^[^ ^8^, ^9^, ^10^, ^11^, ^12^, ^13^, ^14^, ^15^, ^16^, ^17^, ^18^, ^19^, ^20^, ^21^, ^22^ ^]^ To achieve thrombolytic therapy, researchers have loaded micro/nanorobots with thrombolytic drugs and targeted the thrombus site under a magnetic field, increasing the local drug concentration and improving thrombolytic efficiency.^[^ ^23^, ^24^, ^25^ ^]^ Guan et al. conjugated heparinoid‐polymer brushes on the surface of nanorobots, which could realize reversible agglomeration‐free reconfigurations.^[^ ^26^ ^]^ Zhang and colleagues utilized tPA‐anchored nanorobots to dissolve thrombi in submillimeter blood vessels under the guidance of X‐ray imaging and retrieved the nanorobots with an interventional catheter.^[^ ^27^ ^]^ In our previous study, we directly delivered thrombolytic drugs and revealed that the locomotion of nanorobot collectives can guide drug‐targeted diffusion through mass transportation theory.^[^ ^28^ ^]^ The nanorobots could also increase the local flow rate to improve the contact between r‐tPA and the thrombus, thereby enhancing thrombolysis.^[^ ^29^ ^]^ All these studies demonstrate the clinical application prospects of magnetic nanorobots in thrombolysis.

To improve the ability of nanorobots to reach the designated site and more efficiently improve drug delivery, researchers have extensively investigated methods to control magnetic nanorobots in blood vessels and explored their behavior in physiological environments.^[^ ^30^, ^31^, ^32^, ^33^, ^34^, ^35^, ^36^ ^]^ Wu et al. improved the retention of nanorobots in flowing blood by modifying the movement of the nanorobots from rolling to rotating locomotion.^[^ ^37^ ^]^ Zhang et al utilized an interventional catheter to deliver nanorobots and opened balloons to block vasculature and reduce the impact of blood flow on the locomotion of nanorobots.^[^ ^13^, ^27^ ^]^ Magnetic nanorobot collectives can move along the wall of blood vessels, which possesses a much lower shear flow than that in the center of the vessel.^[^ ^38^ ^]^ However, the micro/nanorobots can only rotate and move in the plane due to the magnetic attraction force under rotating permanent magnet systems, which limits the drug transportation efficiency because the fluid disturbance in the vertical direction is limited. Recent studies have shown that the swinging of magnetically controlled cilia arrays can achieve drug transport.^[^ ^39^ ^]^ And it was demonstrated that magnetic torque‐actuated microrobots can more efficiently improve the delivery of drugs under a rotating magnetic field.^[^ ^40^, ^41^ ^]^ It is reasonable to assume that cilia‐mimic array of magnetic colloids could more greatly disturb the fluid by increasing the height of collectives to enhance drug transportation more efficiently under a torque‐force hybrid magnetic field. However, thrombolytic drugs still face the challenge that cannot effectively penetrate into thrombi with abundant platelets and fibrin tissue, further hindering efforts to improve thrombolysis efficiency.

With mechanical cavitation effect, thermal effect, acoustic radiation force and other physical effects, ultrasound combined with microbubbles was utilized to enhance nanoparticles penetration into solid tumors.^[^ ^42^ ^]^ And low‐intensity focused ultrasound combined with microbubbles can also improve thrombolysis.^[^ ^24^, ^43^ ^]^ In this way, ultrasonic energy can be used to burst the microbubbles at the thrombus site, thus accelerating thrombolysis. However, there is a risk of vascular damage due to uncontrolled rupture of contrast microbubbles. And it may produce many thrombus fragments with diameters greater than 10 µm during thrombolysis, which can cause downstream vascular embolism.^[^ ^44^, ^45^ ^]^ Recently, researchers have reported that the physical effect of low‐intensity ultrasound can change the permeability of tissue and enhance the adhesion of gels to skin.^[^ ^46^ ^]^ And the fluid vortex motion induced by acoustic streaming can enhance the contact between drugs and thrombus.^[^ ^47^ ^]^ Therefore, it is reasonable to assume that cilia‐mimic locomotion of magnetic colloidal collectives improves thrombolytic drug transportation and low‐intensity ultrasound increase the permeability of thrombi to achieve penetration of thrombolytic drugs to potentiate thrombolysis. Although promising, the feasibility to actuate ciliary locomotion of magnetic colloidal collectives combined with low‐intensity ultrasound irradiation to enhance thrombolytic drug transportation and penetration still need to be validated in vivo.

**Scheme 1:**
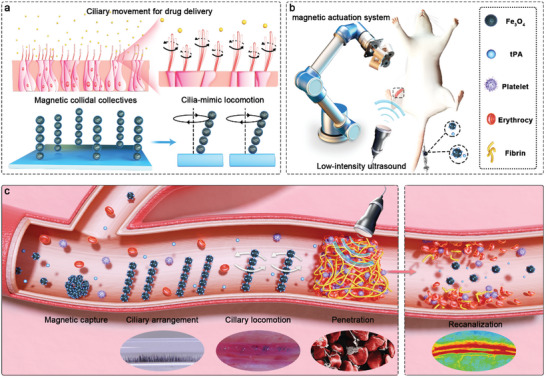
Conceptual overview of the enhanced penetration of thrombolytic drugs by cilia‐mimic locomotion of magnetic colloidal collectives combined with low‐intensity ultrasound. a) Cilia‐mimic arrangement and locomotion of magnetic colloidal collectives. b) The rat femoral vein thrombosis model treated by magnetically actuated colloids and low‐intensity ultrasound. c) Enhanced thrombolytic drug delivery and penetration process by the locomotion of magnetic colloidal collectives, including magnetic target capture, ciliary arrangement and locomotion, and irradiation of low‐intensity ultrasound to achieve fast recanalization.

In this work, we developed an approach to improve the penetration of thrombolytic drugs by the regulating ciliary locomotion of colloidal collectives under a torque‐force vortex magnetic field with low‐intensity ultrasound irradiation (**Scheme** **1**). As a proof of concept, magnetic Fe_3_O_4_ colloids mixed with tPA solution were captured at the thrombus site. In contrast to a torque‐force rotating magnetic field, a torque‐force vortex magnetic field allows magnetic colloidal collectives to align like cilia perpendicular to the rotation plane and perform vortex motion, increasing the transportation of thrombolytic drugs. Simultaneously, low‐intensity ultrasound was applied to enhance drug penetration into the interior of the thrombus and further accelerate thrombolysis by causing fiber breakage and erythrocyte deformation. In vitro thrombolytic experimental results demonstrated that the thrombolysis efficiency increased by 16.3 times compared with that of native tPA treatments. Lastly, a rat model for femoral vein thrombus confirmed that the strategy could realize blood flow recanalization more quickly in vivo by a laser flowmeter. The proposed strategy integrates cilia‐mimic locomotion of magnetic colloidal collectives and low‐intensity ultrasound to improve thrombolytic drug penetration to increase the thrombolysis efficiency, providing a promising pharmacologic thrombolytic therapy approach to realize rapid recanalization by magnetic micro/nanorobotic systems.

## 2 Results

### 2.1 Preparation and Characterization of Magnetic Colloids

To design a permanent actuated system to enhance drug transportation, magnetic Fe_3_O_4_ colloids were first synthesized by a solvothermal method as previously reported. Scanning electron microscopy (SEM) and transmission electron microscopy (TEM) demonstrated that the fabricated magnetic colloids exhibited a homogeneous spherical structure with mesopores (Figure S1, Supporting Information). Elemental mapping and energy dispersive X‐ray (EDX) spectroscopy verified that the magnetic colloid contained only Fe and O (Figures S2 and S3, Supporting Information). The size distribution histogram indicated that the average diameter was 225.9 ± 15 nm (Figure S4, Supporting Information). Magnetic hysteresis curves showed that the colloidal nanoparticles had a saturation magnetization of 68.1 emu g^−1^ in the high magnetic field region of 20000 Oe, which is typical of high‐frequency hysteresis loops (Figure S5, Supporting Information). The nanoparticles could be quickly separated from the dispersed solution by the magnet, revealing a good magnetic response. SEM imaging and TEM imaging demonstrated that the substances absorbed on the colloidal surfaces after the nanoparticles were mixed with the tPA solution (Figure S6, Supporting Information). The calculated drug loading ratio was 7.4% (Figure S7, Supporting Information).

### 2.2 Cilia‐Mimic Locomotion of Magnetic Colloidal Collectives

**Figure 1:**
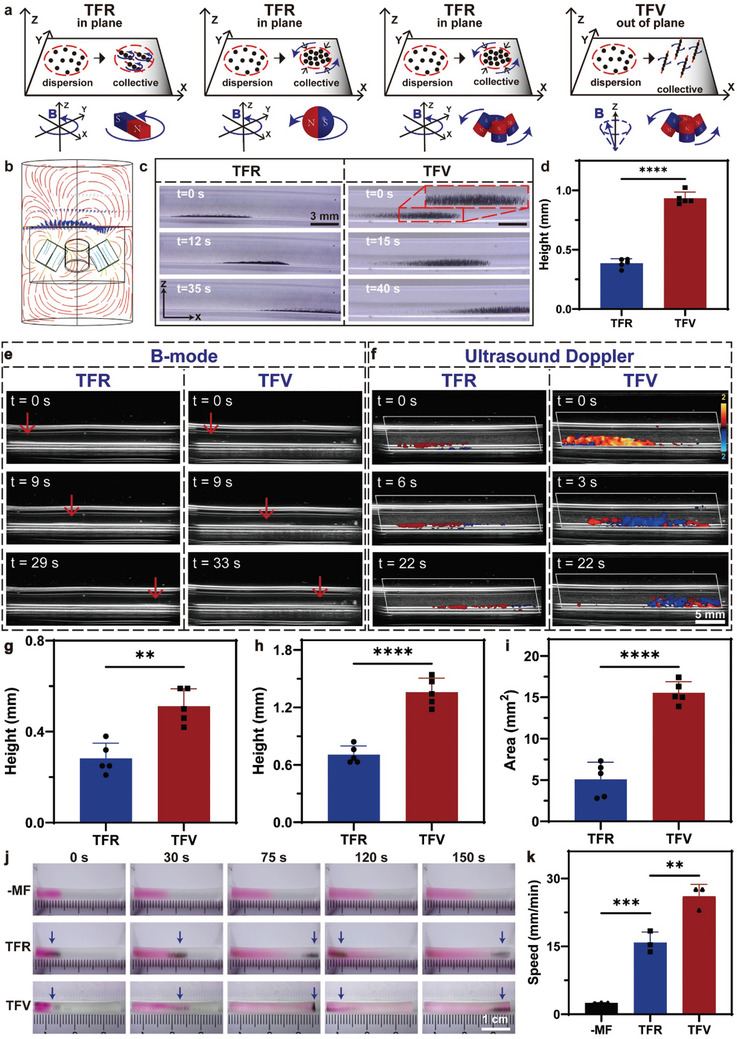
TFV‐MF enhancing ultrasound imaging and drug diffusion. a) Schematic illustrating locomotion of magnetic colloidal collectives under different magnetic field. b) Simulation results showing the magnetic field distribution of the TFV‐MF. c) Snapshot images showing the locomotion of magnetic colloidal collectives from the side view under TFR‐MF and TFV‐MF, respectively. d) Quantitative analysis of the height of the magnetic colloids under TFR‐MF and TFV‐MF, respectively. e) B‐mode ultrasound imaging of the locomotion of magnetic colloidal collectives under different magnetic field. The yellow arrow indicating the magnetic colloids. The red arrow indicating the magnetic colloids. f) The ultrasound color doppler imaging of magnetic colloidal collectives under different magnetic field. Quantitative comparison of the height of the signal enhancement region by the colloidal collectives in ultrasound b‐mode imaging g) and color doppler mode imaging h) under TFR‐MF and TFV‐MF, respectively. i) Quantitative comparison of the area of signal enhancement region by the colloidal collectives in color doppler mode. j) Snapshots showing dye molecules diffusion under the actuation of different magnetic field. The blue arrows indicating the magnetic colloidal collectives. k) Quantitative analysis of diffusion speed of dye molecules under different magnetic field.

To drive the locomotion of magnetic colloids, a rotating permanent magnet has previously been used to generate a torque‐force rotating magnetic field (TFR‐MF) (**Figure** **1**a). The permanent magnet can be cylindrical, rectangular, spherical, or a combination of permanent magnets. With the help of rotating motors, the magnets can simultaneously generate a gradient magnetic field and a rotating magnetic field, which can drive the aggregation and locomotion of magnetic colloids. In the presence of the TFR‐MF, the magnetic colloids are actuated to rotate in a plane due to magnetic attraction. In this study, by adjusting the arrangement of magnets, we designed a magnetic actuation system that could drive nanorobot collectives to perform vortex locomotion. In contrast to TFR‐MF, magnetic colloids can be arranged in a chain shape perpendicular to the plane like cilia and rotate under a torque‐force vortex magnetic field (TFV‐MF). The simulated magnetic field distribution is shown in Figure 1b. Due to the repulsive force of the three magnetic poles, a vertical upward magnetic field line distribution was generated. The magnetic field change was also simulated on the plane (variation distance = 1.5 cm) when the magnets rotated at a frequency of 2 Hz. The center of the vertical magnetic field line distribution also began to undergo periodic changes, which could cause the magnetic colloid chains to undergo vortex motion (Figure S8, Supporting Information).

Then, the cilia‐mimic locomotion of magnetic colloids was conducted in a tube under a TFV‐MF. First, we explored the effects of frequency and strength of the magnetic field on the behavior of cilia‐mimic colloids. As shown in Figures S9 and S10 (Supporting Information), we adopted a rotation frequency of 2 Hz and a variation distance of 2 cm in the following in‐vitro experiments. Under TFR‐MF, the colloids were actuated to aggregate into a swarm on the plane. Under TFV‐MF, the magnetic colloids were arranged in chains perpendicular to the ground like cilia, and vortex motion was observed from the side view (Figure 1c; Video S1, Supporting Information). Quantitative analysis results indicated that magnetic colloidal collectives under TFV‐MF had a higher height than those under TFR‐MF (Figure 1d). Based on the top view, the colloids could form a swarm but not aggregate (Figure S11 and Video S2, Supporting Information). Next, by adjusting the rotation direction and position of the magnetic actuation system, the magnetic colloids were actuated to move in “W”‐shaped and “Y”‐shaped modes under TFV‐MF (Figure S12 and Video S3, Supporting Information).

### 2.3 Enhanced Ultrasound Imaging under TFV‐MF

Subsequently, real‐time ultrasound imaging of the motion of the colloidal collectives was conducted. As shown in Figure 1e, under TFR‐MF, only a small echogenic enhancement was observed along the bottom of the tube because the magnetic colloids rotate in a plane (Video S4, Supporting Information). In contrast, the cilia‐mimic colloidal collective motion under TFV‐MF demonstrated a large range of echo enhancement. In addition, the doppler signals induced by the locomotion of magnetic colloids under TFR and TFV magnetic fields were compared (Figure 1f; Video S5, Supporting Information). During rotation and vortex motion, the colloids and the surrounding blood cells moved in different directions relative to the transducer, creating both red and blue Doppler signals. Under the TFR‐MF, the collective motion was confined to the region close to the edge of the tube wall, with a smaller range of colored signals. However, under TFV‐MF, a larger range of colloid motion along the z‐axis leads to an increase in the area of color signals.

Next, the increase in the ultrasonic signal strength was quantitively analyzed. As shown in Figure 1g, the average height of magnetic colloids in b‐mode under TFV‐MF was greater than that under TFR‐MF. The average height of the color doppler signal induced by the magnetic colloids was 0.71 mm under TFR‐MF and 1.36 mm under TFV‐MF (Figure 1h). The average area of the color doppler signal was 5.1 mm^2^ under TFR‐MF and 15.5 mm^2^ under TFV‐MF (Figure 1i). The average height and area of the doppler signal induced by the motion of magnetic colloids under TFV‐MF were 1.92 times and 3.04 times greater than those under TFR‐MF, respectively. The above results indicated that magnetic colloids under TFV‐MF could significantly enhance ultrasound imaging compared with those under TFR‐MF.

### 2.4 Enhanced Drug Diffusion under TFV‐MF

Given that the cilia‐mimic magnetic colloidal collectives had a greater height under the TFV‐MF, which might lead to greater fluid disturbance, we attempted to verify whether drug diffusion was more quickly increased by the locomotion of magnetic colloids than the locomotion of the TFR‐MF. A rhodamine B dye solution (5 µL, 5 mg mL^−1^) containing the simulated drug was added to a tube filled with normal saline to observe the diffusion rate. As shown in Figure 1j, due to the concentration gradient, the dye in the control group without any intervention diffused slowly to the other end of the tube, and the front end of the dye color diffused ≈7 mm in 150 s. In the TFR and TFV groups, magnetic colloid solution (50 µL, 5 mg mL^−1^) was added to the tube. After the dye solution was added, TFR and TFV magnetic fields were applied with a rotating frequency of 2 Hz. The magnetic colloids were moved to the other end of the tube under a magnetic field. The results indicated that dye molecules could be guided to diffuse quickly based on mass transport theory. More dye molecules were transported when the magnetic colloids arrived at the end of the tube under TFV‐MF than under TFR‐MF at 75 s. The quantitative analysis results indicated that the average diffusion speeds of the dye molecules were 2.5, 15.8, and 26.1 mm min^−1^ without a magnetic field and with TFR‐MF and TFV‐MF, respectively (Figure 1k). The drug diffusion rate in the TFV‐MF group was 65% greater than that in the TFR group and 10.4 times greater than that in the free diffusion group.

### 2.5 Capture of Magnetic Colloids under TFV‐MF

**Figure 2:**
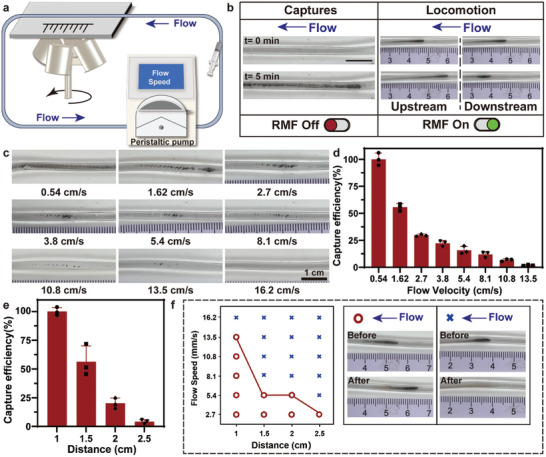
Capture of the magnetic colloids under TFV‐MF in fluid environment. a) Schematic of the capture and actuation of magnetic colloids in the fluid environment under TFV‐MF. b) Snapshot images showing the capture and locomotion of magnetic colloids (upstream and downstream) under TFV‐MF. Scale bars, 1 cm. c) Representative optical images of colloids captured at different flow velocity. d) Quantification of capture efficiency of magnetic colloids at different flow velocity. e) Quantification of capture efficiency of magnetic colloids in different distance separating from the magnetic actuation system. f) The phase diagram showing the motion magnetic colloidal collectives actuated by TFV‐MF at different flow speed. The right optical images showing the possible motion pattern states.

To increase the penetration of thrombolytic drugs, magnetic colloids must first be captured at the thrombus site, followed by vortex locomotion. Considering the effect of blood flow on the locomotion of magnetic colloids, a fluid piping system was used to simulate the flow environment (**Figure** **2**a). The magnetic actuation system was deployed under the pipe to capture and drive the locomotion of magnetic colloids. In the capture procedure, the rotating motor was turned off. Then, the rotating motor was turned on, causing the magnetic colloids to perform ciliary locomotion and move upstream and downstream (Figure 2b). The capture efficiency of magnetic colloids at different flow rates and their ability to overcome the flow fluid were explored under TFV‐MF (Figure 2c). The amount of captured magnetic colloids decreased with increasing flow velocity. When the flow velocity reached 0.54 cm s^−1^, the captured magnetic colloids were filled with the tube. When the flow velocity increased to 13.5 cm s^−1^, which is equivalent to the normal velocity of venous blood flow in the lower limbs, some colloids remained in the tube. The results of the quantitative analysis indicated that the capture efficacy was 22% to 12% at flow velocities ranging from 3.8 to 8.1 cm s^−1^, which is equivalent to the velocity of embolic venous blood flow in the lower limbs (Figure 2d).

As the distance increases, the magnetic field intensity decays rapidly. We further explored the capture efficiency of magnetic colloids at different distances. With increasing distance, the amount of captured magnetic colloids gradually decreased (Figure S13, Supporting Information). The quantitative analysis results indicated that magnetic colloid capture is possible as far as 2.5 cm away at a flow velocity of 2.7 cm s^−1^ (Figure 2e). Then, the rotating motor was turned on, which caused the captured colloids to form collectives and achieve vortex movement in the upstream and downstream directions. The ability of the magnetic colloids to overcome the flow fluid state of colloids under TFV‐MF was also investigated at different flow speeds and distances (Figure 2f). As the flow velocity and distance increased, the ability of magnetic colloids to move under a magnetic field decreased. Compared to the velocity that the robot could overcome when capturing, the velocity that magnetic colloids could overcome when moving was one order of magnitude lower. Notably, the blood flow near the thrombus was interrupted, and the flow rate decreased to a very low level, which facilitated the locomotion of magnetic colloids. The above experimental results indicated that the TFV‐MF could capture and regulate the movement of magnetic colloids in a fluid flow environment.

### 2.6 Low‐Intensity Ultrasound Increases Drug Penetration

**Figure 3:**
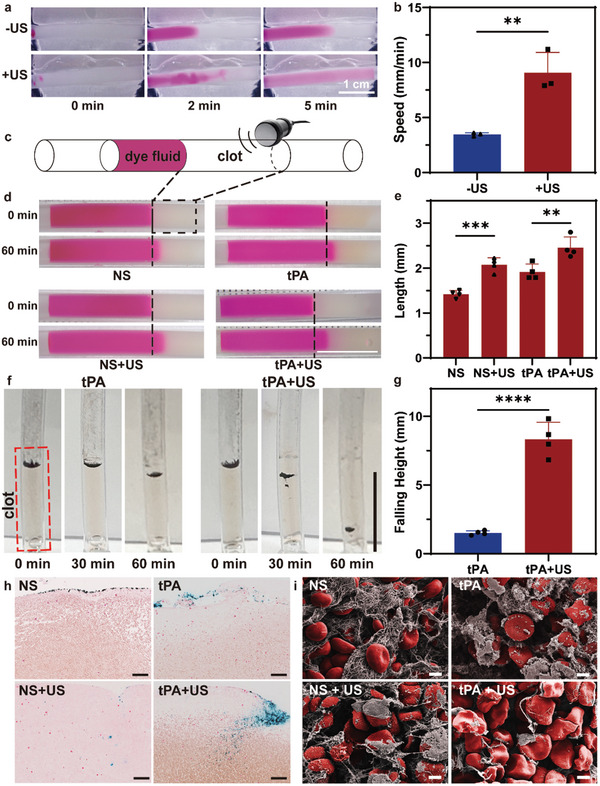
Low‐intensity ultrasound enhancing drug diffusion and penetration. a) Dye molecules diffusion enhanced by ultrasound. b) Quantitative evaluation of the diffusion speed of dye molecules reaching the end of the tube. c) Schematic diagram of ultrasound enhancing dye fluid penetration. d) The dye fluid penetration under different treatment. Scale Bar, 1 cm. e) Quantification of the length change of dye fluid within 60 min. f) The process of magnetic colloids penetrating into clots under different treatment. Scale Bar,1 cm. g) Quantification of the falling height of magnetic colloids with ultrasound irradiation. h) Prussian blue staining showing magnetic colloids in clot slice. Scale Bar, 50 µm. i) Representative SEM images showing the morphological structure of clots under different treatments. Scale Bar, 2 µm.

To increase drug penetration into the thrombus and improve thrombolytic efficiency, low‐intensity ultrasound was used. First, the ability of low‐intensity ultrasound to increase drug diffusion was explored. The color change of the tube filled with normal saline was recorded after dye solution (5 mg mL^−1^, 5 µL) was added with low‐intensity ultrasound irradiation (**Figure** **3**a; Video S6, Supporting Information). The dye molecules diffuse slowly to the other end in the control group. And in the ultrasound treatment group (0.5 W cm^−2^), the color of the tube changed to the dye color within 5 min. Quantitative analysis revealed that the average diffusion speed of dye molecules was 9.1 mm min^−1^ with ultrasound irradiation, which was approximately three times greater than that in the control group (3.5 mm min^−1^) (Figure 3b).

To validate the ability of ultrasound to enhance drug penetration, a transparent clot model was fabricated with blood plasma (Figure 3c). A dye solution mixed with different thrombolysis agents was added, and the cells were subjected to low‐intensity ultrasound. As shown in Figure 3d, the edge of the dye solution gradually penetrated into the transparent clot in 60 min. In the normal saline (NS) group, the dye solution penetrated slightly into the transparent blood clot. After ultrasound was applied (0.5 W cm^−2^, 60 min), the dye penetrated into the clot farther, which was also observed for the tPA group. Due to the dual effects generated by diffusion of the dye molecules and tPA thrombolysis, the change in dye infiltration of the transparent blood clot was more pronounced than those generated by individual effects. Changes in the dye column length were measured to quantify the effect of low‐intensity ultrasound treatment on increasing tPA penetration into the thrombus (Figure 3e). The average length changes of the dye columns with different treatments were 1.4, 2.1, 1.9, and 2.5 mm. In both the normal saline group and the tPA group, the infiltration length of dye molecules in the group treated with ultrasound was significantly greater than that in the group without ultrasound irradiation.

Additionally, we performed ultrasound‐enhanced drug penetration experiments in a whole blood clot model. The Cy5.5 fluorescent dye was mixed with tPA and injected into whole blood thrombi. After ultrasound treatment, the change in the length of the fluorescent column was significantly greater than that of the native tPA group (Figure S14, Supporting Information). These results indicated that low‐intensity ultrasound can increase drug penetration into the thrombus.

**Figure 4:**
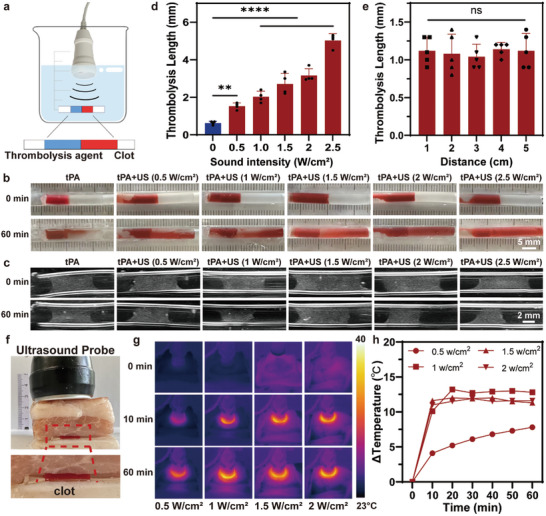
Assessment of enhancing dissolution efficiency of blood clots by ultrasound with different parameters in vitro. a) Schematic diagram of in‐vitro experiment of ultrasound‐enhanced thrombolysis. Snapshots b) and ultrasound images c) of the change of clots treated with different sound intensity in 60 min. d) Quantitative evaluation of thrombolysis length treated with different sound intensity using water as medium. e) Quantitative evaluation of thrombolysis length treated by ultrasound (0.5 W cm^−2^) at different distances varying from the probe. f) Schematic diagram of ultrasound enhancing thrombolysis using pork as medium. g) Thermographs showing the surface temperature change of the pork treated by ultrasound with different sound intensity. h) The corresponding temperature rise curves treated by ultrasound with different sound intensity.

We further explored whether low‐intensity ultrasound could enhance the penetration of magnetic colloids into the thrombus. To obtain better observations, we added a mixture of magnetic colloid (50 µL, 5 mg mL^−1^) and tPA solution (20 µg mL^−1^) to the tube with a transparent clot. Due to gravity, the nanorobots move to the surface of the clot first. After 60 min, the nanorobots almost stayed at the same position above the clot in the saline group without any treatment, demonstrating that a transparent clot was firmly established (Figure S15, Supporting Information). In the group injected with the magnetic colloid dispersion mixed with tPA solution, a slow decrease in the magnetic colloid position was observed; however, the colloids remained on the surface of the thrombus (**Figure** **4**f; Figure S16, Supporting Information), suggesting that the drug‐mediated thrombolysis process was progressive. In the ultrasound treatment group (0.5 W cm^−2^, 60 min), the magnetic colloids fell rapidly, and nanorobots were observed inside the transparent blood clot. The average falling height in the native tPA treatment group was 1.5 mm, while with ultrasound treatment, the average falling distance reached 8.3 mm (Figure 4g).

Next, we performed experiments to examine ultrasound‐enhanced magnetic colloid penetration in whole blood thrombi. After treatment with different agents, the thrombi were collected for hematoxylin‐eosin (HE) and Prussian blue staining. As shown in Figure 4h, more Prussian blue‐stained magnetic colloids were observed in the interior of the thrombi in the ultrasound treatment group, and in the thrombi without ultrasound, most of the particles were distributed on the surface (Figure S17, Supporting Information). Dppa‐labeled magnetic colloids were also used to evaluate the effect of ultrasound‐enhanced penetration into thrombi. The fluorescence images demonstrated that more magnetic colloids penetrated into the clots after low‐intensity ultrasound treatment (Figure S18, Supporting Information).

In addition, biological SEM images of the thrombi were obtained to explore the physical effect of ultrasound on the fibrin network. As shown in Figure 3i, the deformed RBCs and fibrin were broken into fragments on the surface of clots after ultrasound irradiation, while red blood cells (RBCs) were encompassed by dense fibrin on the surface of the clots treated with NSs (Figure S19, Supporting Information). In the native tPA group, partial fibrin lysis occurred, and the network structure of the fibrin disappeared. In the group treated with tPA and ultrasound (0.5 W cm^−2^, 60 min), the morphology of red blood cells exhibited several changes, including wrinkling, while fibrin lysis occurred almost exclusively. Therefore, it was speculated that low‐intensity ultrasound has a destructive effect on the fibrin network, enhancing the penetration of tPA into the thrombus and increasing thrombolysis efficiency. These results verified that ultrasound treatment could improve the permeability of tPA into the clot as well as enhance fibrinolysis.

### 2.7 Low‐Intensity Ultrasound Improves Thrombolysis Efficiency In Vitro

Next, to investigate the improved thrombolysis efficiency by the low‐intensity ultrasound, various parameters were explored in vitro. tPA solution (20 µg mL^−1^) was added to the tube with a red clot, and low‐intensity ultrasound was applied with different sound intensities. Ultrasound imaging was used to measure the length change of the clots. The results indicated that the length of thrombolysis increased with increasing ultrasound power (Figure S20, Supporting Information). Considering that heating induced by the ultrasound probe may affect thrombolysis, water was used as the medium for ultrasound treatment to eliminate the influence of thermal effects (Figure 4a). After ultrasound treatment at 0, 0.5, 1, 1.5, 2, and 2.5 W cm^−2^ was applied for 60 min, the changes in the thrombus were recorded by a digital camera and an ultrasound image, respectively (Figure 4b,c). The results showed that the thrombolytic efficiency of ultrasound therapy was greater than that of pure tPA treatment, even without ultrasound thermal effects. The changes in the length of the thrombus on ultrasound were measured and quantitative analysis was performed, revealing that low‐intensity ultrasound could significantly enhance thrombolysis efficacy at a sound intensity of 0.5 W cm^−2^ (Figure 4d). In addition, thrombolysis length increased with increasing sound intensity. Next, the activity of tPA was evaluated with different ultrasound sound intensities. Changes in optical density (OD) at 405 nm were used to assess the enzyme activity of tPA over the corresponding reaction time. The curves showed that enzyme activity increased with low‐intensity ultrasound irradiation compared to that of pure tPA (Figure S21, Supporting Information). In addition, the values increased with increasing sound intensity. This finding verified that low‐intensity ultrasound is effective in increasing the enzyme catalytic activity of tPA, thereby potentiating pharmacological thrombolysis.

As the depth of blood vessels inside the human body varies from the surface of the body, different distances between the ultrasound probe and thrombus were also investigated. The results demonstrated that there was no significant difference in thrombolysis length when the ultrasound probe was placed at different distances from 1 to 5 cm, indicating that the increase in thrombolysis efficiency by low‐intensity ultrasound did not decrease obviously with increasing distance (Figure 4e; Figure S22, Supporting Information).

Because ultrasonic energy will decay after passing through the tissue, we further validated the enhanced thrombolytic efficiency of low‐intensity ultrasound through a 2 cm thick pork. Thrombi were prepared in the tube using the same procedure and were placed under the pork (Figure 4f). It was observed that low‐intensity ultrasound could also increase the thrombolysis efficiency compared with that of pure tPA treatment when pork was used as the medium (Figure S23, Supporting Information). With increasing ultrasound intensity, a similar increase in efficiency was observed in the quantitative evaluation of thrombolysis length. Considering that the heat generated by the probe may damage the body surface, the temperature change in the skin surface was monitored by an infrared camera (Figure 4g). With increasing power intensity, a greater temperature increase was observed at 60 min. Quantitative analysis revealed that the temperature of the pig skin increased by 7.8 °C at 60 min when the ultrasound intensity was 0.5 W cm^−2^ (Figure 4h). When the ultrasound intensity was increased to 1 W cm^−2^, the temperature of the pig skin increased by more than 10 °C in 10 min and reached an equilibrium temperature increase of 12.8 °C. A similar temperature increase was observed when the applied ultrasound intensity was 1.5 and 2 W cm^−2^. Given the temperature tolerance of the human body surface, a sound intensity of 0.5 W cm^−2^ was chosen for the subsequent experiment.

### 2.8 Cilia‐Mimic Locomotion of Magnetic Colloids under Low‐Intensity Ultrasound for Thrombolytic Therapy

**Figure 5:**
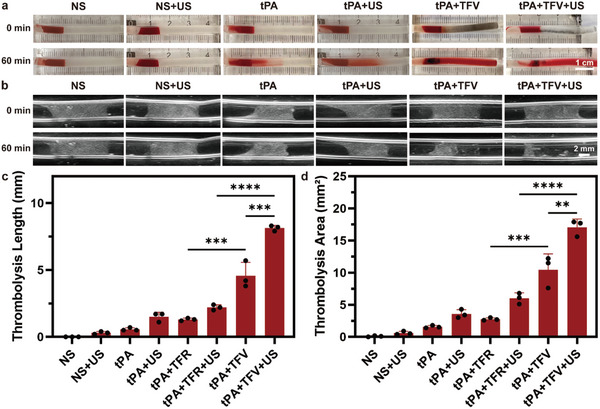
In vitro thrombolysis by ultrasound enhancing torque‐force driven colloidal collectives. a) Representative snapshot of clots after being treated by NS, NS+US, tPA, tPA+US, tPA+TFV, tPA+TFV+US for 60 min. Scale bars, 1 cm. b) Representative ultrasound image of clots after being treated by NS, NS+US, tPA, tPA+US, tPA+TFV, tPA+TFV+US for 60 min. Scale bars, 2 mm. Quantification of thrombolysis length c)and area d)of the clots under different treatments.

Next, we evaluated the thrombolysis efficiency of cilia‐mimic locomotion of magnetic colloids combined with low‐intensity ultrasound. The clots in the tubes were treated with NS, NS+US, tPA, tPA+US, tPA+TFR, tPA+TFR+US, tPA+TFV, or tPA+TFV+US for 60 min, and the changes in thrombi were recorded by digital camera and ultrasound imaging. As shown in **Figure** **5**a, compared with those in the non‐ultrasound group, more thrombi were dissolved in the low‐intensity ultrasound group (0.5 W cm^−2^). The tPA+TFV+US group achieved the maximum thrombolytic length. The ultrasound image verified the changes in thrombi lengths treated by different groups (Figure 5b; Figure S24, Supporting Information). After 60 min of treatment, an average thrombolysis length of 8.1 mm was observed in the tPA+US+TFV group, whereas the average thrombi lengths of native tPA, tPA+US, and tPA+TFV were 0.5, 1.5, and 4.6 mm, respectively (Figure 5c). The thrombolysis area was also calculated and compared. The tPA+US+TFV group achieved an average thrombolysis area of 17.1 mm^2^, while the native tPA, tPA+US, and tPA+TFV groups achieved average thrombolysis areas of 1.6, 3.6, and 10.4 mm^2^, respectively (Figure 5d). Compared with previous thrombus treatment by tPA+TFR, thrombolysis efficiency was significantly greater in the tPA+US+TFV group. The thrombolysis efficiency of the tPA+US+TFV group was 16.2 times of the native tPA group in terms of thrombolysis length and 10.7 times in terms of thrombolysis area. All these results indicated that the thrombolysis efficiency could be notably improved by ultrasound‐enhanced torque‐force‐driven cilia‐mimic colloidal collectives.

### 2.9 Evaluation of Thrombolysis Efficacy in a Rat Model for Femoral Vein Thrombi

**Figure 6:**
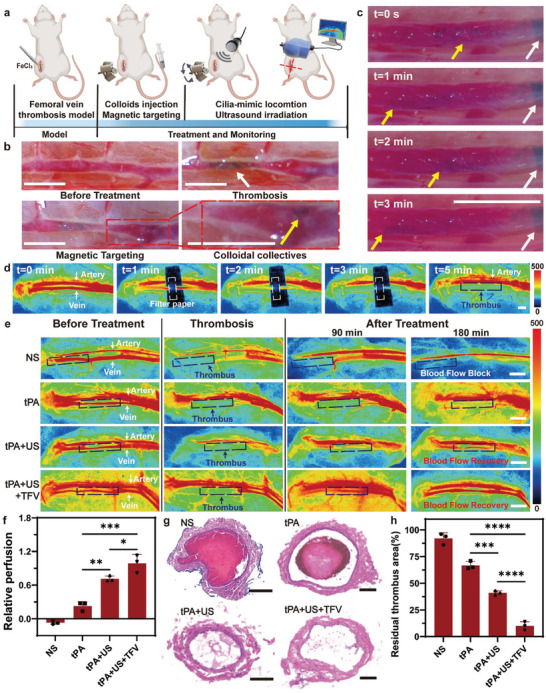
Evaluation of thrombolysis efficacy in a rat mode with femoral vein thrombi. a) Schematic diagram of the thrombolysis treatment of rat femoral venous thrombus in vivo. b) Operating microscope images depicting thrombus formation and magnetic targeting of colloids. Scale bar, 1 mm. c) Operating microscope images illustrating the intravenous cilia‐mimic locomotion of colloids under TFV‐MF. The yellow arrows indicating colloidal collectives and the white arrows indicating thrombus. d) Blood flow images monitored by laser doppler blood flow meter indicating thrombus formation. Scale bar, 2 mm. e) The blood flow change in rat femoral vein from the NS, tPA, tPA+US, tPA+US+TFV groups at different time. Scale bar, 2 mm. f) Quantification of blood perfusion recovery treated with different groups. (n = 3). g) Representative histological analysis of the femoral vein venous after treatment with NS, tPA, tPA+US, tPA+US+TFV, respectively. Scale bar,100 µm. h) Quantification of the residual thrombus area (%vein lumen) in femoral vein under different treatment (n = 3).

To investigate the in vivo thrombolysis efficacy of cilia‐mimic locomotion of magnetic colloids combined with low‐intensity ultrasound, a femoral vein thrombus rat model was established (Figure S25, Supporting Information). As shown in **Figure** **6**a, a femoral vein was pretreated with FeCl_3_ to form a thrombus, and then magnetic colloid solution mixed with tPA was injected via the tail vein. After that, the magnetic actuation system without rotation was applied to capture the magnetic colloids at the thrombus site for 5 min. Then, a TFV magnetic field was applied to drive the magnetic colloid to move locally. Moreover, low‐intensity ultrasound was used to treat the thrombus for 180 min. During this procedure, laser doppler flowmetry was used to monitor changes in blood flow. Compared with those of the normal section, the area of the femoral vein treated with FeCl_3_ filter paper became darker and more stenotic. After magnetic targeting, aggregation of magnetic colloids could be observed in the vessel close to the thrombus (Figure 6b). Under TFV magnetic field, the colloids were arranged like cilia and then actuated to perform vortex locomotion (Figure 6c; Video S7, Supporting Information).

During the treatment, laser Doppler flowmetry indicated that the blood flow was gradually interrupted, confirming that thrombus formation was successful (Figure 6d). Then, TFV magnetic field‐actuated colloidal collectives combined with low‐intensity ultrasound were used to treat the thrombus. As shown in Figure 6e, the pure tPA group showed only a slight increase in blood flow after treatment for 180 min. In comparison, blood perfusion was better restored with low‐intensity ultrasound assistance. The tPA+US+TFV group had the best thrombolytic effect and completely recanalized blood flow (Figure S26, Supporting Information). This result was consistent with the morphological changes in the femoral vein of each treatment group observed under a surgical microscope (Figure S27, Supporting Information). The quantitative analysis showed that blood flow increased from the initial value in all treatment groups except the control group (Figure 6f). The tPA+US+TFV group achieved the best relative recovery of blood flow, with a mean perfusion recovery of ≈99%, which was greater than the 71% in the US+ tPA group and the 29.3% in the pure tPA group. Moreover, the thrombus area (percentage of vein lumen) was quantified by measuring the cross‐sectional area of the thrombus and vein in the histologic images (Figure 6g; Figure S28, Supporting Information). The tPA+US+TFV treatment group achieved the best thrombolysis effect, with the smallest residual thrombus area (≈10%), whereas the thrombus area in the NS, tPA, and tPA+ US groups remained 92%, 66.7%, and 41%, respectively. All these results verified that TFV magnetic field‐actuated magnetic colloid combined with low‐intensity ultrasound could enhance thrombolysis efficiency in vivo.

### 2.10 Safety Assessment of the Thrombolysis System

The in vitro cytocompatibility of the nanoparticles was investigated by evaluating the viability of human umbilical vein endothelial cells (HUVECs). The cells were incubated with different concentrations of Fe_3_O_4_ solution, ranging from 0 to 800 µg mL^−1^. No obvious cytotoxicity was observed after 24 h of incubation, even at high concentrations, indicating that these nanoparticles exhibit relatively good compatibility (Figure S29, Supporting Information).

In addition, to evaluate the biosafety of the thrombolysis system in vivo, an Fe_3_O_4_ solution (100 µL, 1 mg mL^−1^) was intravenously administered to the SD rats. After 1 day and 7 days of treatment, the major organs were collected for pathological histological analysis. The histological images showed no obvious organ damage or inflammatory lesions in the short‐term or long‐term periods (Figure S30, Supporting Information). Moreover, serum biochemistry assays and complete blood panel tests were conducted. There were no obvious differences in any of the measured indicators among the three groups, suggesting that the magnetic colloids exhibited good biocompatibility (Figure S31, Supporting Information). Moreover, the hemolysis analysis results demonstrated that a high concentration of magnetic colloids did not cause significant hemolysis (Figure S32, Supporting Information). All these results showed that the magnetic colloids exhibited good biocompatibility and biosafety. To verify the safety of low‐intensity ultrasound, the vessels were obtained after low‐intensity ultrasound irradiation for 3 h. As shown in Figure S33 (Supporting Information), the pathological section indicated no obvious damage of vascular endothelium was found after low‐intensity ultrasound irradiation.

## 3 Discussion

We have developed a permanent magnetic actuation system to drive magnetic colloidal collectives arrange align vertical to the plane and conduct cilia‐mimic locomotion, together with low‐intensity ultrasound to improve the penetration of thrombolytic drugs. Under TFV magnetic field, cilia‐mimic magnetic colloidal collectives with greater height more greatly disturbed the surrounding fluid in space to enhance drug diffusion more quickly, and notably increased ultrasound imaging. In addition, low‐intensity ultrasound was applied to further increase drug infiltration into the thrombus by causing fiber breakage and erythrocyte deformation. In vitro thrombolytic experimental results demonstrated that the thrombolysis efficiency increased by 16.3 times compared with that of native tPA treatments. In vivo rat model for femoral vein thrombus experimental results confirmed that the approach could realize blood flow recanalization more quickly by a laser flowmeter.

Magnetic nanorobot collectives have been used for targeted delivery of thrombolytic drugs to improve thrombus therapy and show promise for clinical translation. In addition to increasing the local concentration of thrombolytic drugs, the locomotion of magnetic nanorobot collectives can induce changes in the local flow velocity, enhance the diffusion of drugs and improve contact with the thrombus surface to further improve thrombolytic efficiency.^[^ ^28^ ^]^ Recently, magnetic control systems based on rotating permanent magnets have been favored by researchers due to their many advantages, including an open operation space, the ability to combine DSA, ultrasound and other imaging equipment, and strong magnetic field strength; thus, these systems have been used for thrombus treatment, aneurysm embolization, vascular embolization, etc.^[^ ^9^, ^13^, ^27^ ^]^ However, the gradient magnetic field generated by the permanent magnet attracts the micro/nanorobots to the blood vessel wall, and they can only rotate and adhere to the wall; as a result, the disturbance to the fluid in three‐dimensional space is limited. In this study, the custom designed permanent magnetic actuation system could actuate magnetic colloidal collectives arranged vertical to the blood vessel wall like cilia and conduct vortex motion (Figure 1C). Compared with the TFR magnetic field generated by the rotating magnet, cilia‐mimic magnetic colloidal collectives had a greater height under the TFV magnetic field, more greatly disturbed the surrounding fluid in space, and significantly enhance drug diffusion more quickly (Figure 1J). Beyond this, under TFV‐MF the magnetic colloidal collectives could form a larger range and there are gaps between particle chains, which may be helpful in delivering thrombolytic drugs than rotating swarms.

Due to the small size of magnetic colloids, real‐time monitoring has been a challenge that researchers continue to address. Ultrasound imaging has been widely used for real‐time image monitoring in previous studies due to its many advantages, including real‐time imaging, lack of radiation and deep penetration depth. As the magnetic colloids rotate in a plane under the rotating permanent magnetic system, only a small echogenic enhancement can be observed. In contrast, the cilia‐mimic collective motion under TFV‐MF significantly enhanced ultrasound b‐mode imaging and color doppler imaging (Figure 1E,F).

The locomotion of magnetic colloids can increase the local concentration of drugs at the site of the thrombus and improve the contact between drugs and the thrombus; however, further improvements in thrombolytic efficiency are limited because thrombolytic drugs cannot quickly and easily enter the interior of the thrombus. The physical effects of ultrasound can enhance the penetration of drugs into tissues.^[^ ^46^ ^]^ In this study, the in vitro experimental results indicated that low‐intensity ultrasound could enhance the diffusion and penetration of drugs into the thrombus through acoustic flow effects. After ultrasound irradiation, more fibrin in the thrombus tissue was broken and red blood cells became deformed with larger gaps, suggesting that it was feasible for thrombolytic drugs to enter the interior area (Figure 3I). In vitro thrombolysis experiments verified that thrombolysis efficiency could be significantly improved after ultrasound irradiation was applied, even excluding the thermal effect of the probe. Notably, the power intensity used in this study was 0.5 W cm^−2^, which is very low. This was performed to avoid the thermal effects caused by prolonged operation of the probe, which can damage the skin. In the future, low heat generation and wearable ultrasound probes can be used for clinical treatment.

We validated the thrombolytic efficacy of this system architecture in vitro and in vivo in an animal model. Effective thrombus dissolution was achieved in vitro and in a rat animal thrombus model. There still exist some limitations of strategy from bench to clinic. Due to the limited magnetic field strength, low blood flow vascular environment was adopted to validate the strategy. Thus, increasing the magnetic field strength is necessary to drive colloids motion in high‐flow‐velocity environments. And the magnetic actuation system and ultrasound therapy device should be integrated to achieve precise treatment of thrombus synergistically. In addition, optimizing the surface properties of magnetic colloids is helpful to increase the drug loading capacity. And surface modification of magnetic colloids is expected to increase biosafety and enhance dispersibility, reducing the risk of vascular blockage.

## 4 Conclusion

In summary, we have proposed an approach to enhance the penetration of thrombolytic drugs by the cilia‐mimic locomotion of colloidal collectives under a torque‐force vortex magnetic field combined with low‐intensity ultrasound irradiation. The in vitro and in vivo experimental results demonstrated that this approach could significantly improve thrombolysis efficiency. The vertical arrangement of magnetic colloidal collectives could also enhance ultrasound imaging. In this system, magnetic colloids can achieve “target capture” and “cilia‐mimic locomotion” to guide drug diffusion. Simultaneously, low‐intensity ultrasound was applied to further enhance drug infiltration by producing fiber breakage and erythrocyte deformation. It is anticipated that cilia‐mimic magnetic actuated colloidal collectives locomotion with low‐intensity ultrasound would notably enhance thrombolytic drug transportation for thrombus therapy, demonstrating promising clinical translation prospect. This work also provides a common platform for targeting enhanced drug penetration with magnetic micro‐nano robots in treating other diseases, such as bladder cancer.

## 5 Experimental Section

### Preparation of Magnetic Colloids Mixed with tPA

The hydrothermal synthesis of magnetic Fe_3_O_4_ colloids was performed according to a previously reported protocol.^[^ ^48^ ^]^ The reactants FeCl_3_·6H_2_O (1.35 g) and ammonium acetate (3.6 g) were sequentially added to a 30 mL ethylene glycol, followed by continuous magnetic stirring for 4 h. The obtained mixture was transferred into a 30 mL teflon‐lined stainless‐steel autoclave. For hydrothermal reactions, the autoclave was sealed and heated at 200 °C for 24 h. The Fe_3_O_4_ colloids were then collected via magnetic attraction after the autoclave's temperature dropping to room temperature. They were washed three times using absolute ethanol and re‐suspended in PBS solutions for storage.

The tPA powder (Activase, Genetech, Inc., USA) was dissolved in normal saline to a final concentration of 0.1 mg mL^−1^ in 2 mL. Then, 8 mL of magnetic colloidal solution was added to the tPA solution, and the mixture was stirred for 1 h at room temperature. Finally, the collected Fe_3_O_4_ colloids mixed with tPA solution were stored in a refrigerator at 4 °C. The drug loading percentage was calculated as follows:

(1) $$\mathrm{drug}\;\mathrm{loading}\;\mathrm{ratio}\;\left(\%\right)=\frac{\mathrm{content}\;\mathrm{of}\;\mathrm{total}\;\mathrm{tPA}-\mathrm{content}\;\mathrm{of}\;\mathrm{free}\;\mathrm{tPA}}{\mathrm{content}\;\mathrm{of}\;\mathrm{total}\;\mathrm{tPA}}$$

The free tPA refers to the tPA that remains within the supernatant after drug loading. The content of the tPA loaded was tested by a bicinchoninic acid (BCA) protein assay kit.

### Characterization of Magnetic Colloids

The morphology and elemental distribution of the Fe_3_O_4_ colloids were observed using a scanning electron microscope (Sigma 300, Zeiss, Germany) and a transmission electron microscope equipped with high‐angle annular dark‐field imaging (JEM‐2100F, JEOL, Japan), respectively. The resultant SEM‐EDX elemental maps were used to plot the abundance of iron and oxygen in the samples. Diameters were measured manually from the photographs by using ImageJ software, and at least 200 randomly selected nanoparticles were counted. The magnetic properties of the nanorobots were estimated using a vibrating sample magnetometer (7404, Lake Shore Cryotronics, Westerville, OH, USA).

### Magnetic Actuation System

The magnetic actuation system in this study was a customized device that integrates quadruple magnet modules (30 mm‐diameter N52‐graded NdFeB) on a rotatory motor. The inclination angle of the four magnets along the z‐axis was 30°, among which the N pole of three magnets was upward and the N pole of one magnet was downward. The rotating permanent magnetic actuation motor were installed on a robotic arm with six degrees of freedom to realize free position regulation.

### Ultrasound Imaging of Cilia‐Mimic Locomotion of Colloidal Collectives

The magnetic colloidal solution (50 µL, 1 mg mL^−1^) was injected into a tube filled with normal saline, and then TFR‐MF or TFV‐MF (2 Hz) was applied. The motion of the magnetic colloidal collectives was recorded by ultrasound imaging (frequency: 12.5–23.0 MHz; depth: 2.0 cm; gain: 60; frequency rate: 12; dynamic range: 120, L20‐5 U; Mindray Resona9 Pro, Mindray, China). For color Doppler ultrasound imaging, a colloidal dispersion (50 µL, 1 mg mL^−1^) was added to a tube filled with fresh rabbit anticoagulated blood. The blood flow signal was formed by the perturbation of the surrounding blood cells, and color Doppler was used to observe the movement of the colloidal collectives.

### Dye Molecular Diffusion Assessment

The magnetic colloidal solution (50 µL, 1 mg mL^−1^) was injected into a tube filled with normal saline. Then, 5 µL of Rhodamine B dye solution (5 mg mL^−1^) was slowly injected from the open end of the tube using a 50 µl syringe to prevent the dye from rapidly diffusing during the initial period. TFR‐MF and TFV‐MF were applied to regulate the motion of the nanorobot collective toward the other end of the tube.

### Capture of Colloidal Collectives and Modulation of Motion

The magnetic colloids were captured in a fluid environment by a peristaltic pump and plastic tube with an inner diameter of 3 mm. The colloidal solution (100 µL, 5 mg mL^−1^) was injected by a syringe, and the solution moved with an aqueous flow. In this procedure, the rotating motor was turned off. When the magnetic colloids passed through the tube, they were captured by the magnetic attraction force. The capture efficiency of TFV‐MF was investigated at different flow speeds (0.54, 1.62, 2.7, 3.8, 5.4, 8.1, 10.8, 13.5, and 16.2 cm s^−1^) and at different distances (d = 1, 1.5, 2, and 2.5 cm). The capture efficiency at different speeds was obtained by calculating the ratio of image pixels of colloids captured to that at minimum speed using ImageJ software.

After being captured, the colloids were first regulated to move against the flow and then regulated to move along with the flow. The motion of the retained colloids was recorded by an operating microscope. The effects of varying relative distances (d = 1, 1.5, 2, 2.5 cm) and flow velocities (v = 2.7, 8.1, 13.5, 16.2 mm s^−1^) on the motion of the colloids were investigated.

### Fabrication of the Clot Model

A transparent clot model was fabricated in the tube. Rabbit fresh blood was centrifuged at 5000 rpm for 10 min to obtain plasma. Eighty microliters of plasma were mixed with 12 µL of thrombin solution (Solarbio, 1000 U mL^−1^), injected into an infusion tube, sealed at one end, and maintained at 25 °C for 30 min to generate a firm clot. A red clot model was generated by mixing 80 µL of fresh blood with 12 µL of thrombin.

### Ultrasound Enhances Drug Penetration In Vitro

Liquids for different treatment groups were prepared as follows: Rhodamine B dye was dissolved in normal saline, and the dye was dissolved in tPA solution. For each of the above groups of liquids, the final concentration of tPA was set to 20 µg mL^−1^. In the ultrasound treatment group, the ultrasound probe was submerged under the water surface and placed directly 2 cm above the tube. After ultrasound treatment for 1 h (0.5 W cm^−^^2^), the position of the front end of the dye solution was determined to quantify the penetration of the dye molecules into the inside of the clot. The change in the length of each position was measured by ImageJ software.

Then, magnetic colloids (5 mg mL^−1^) mixed with tPA solution (50 µL, 20 µg mL^−1^) were added to tubes with transparent clots. The tubes were placed vertically and treated by ultrasound (0.5 W cm^−^^2^) for 1 h. The distance between the probe and the tube was 0.5 cm. The falling height of the colloids was defined as the change in position of the bottom‐most end from the initial position after the ultrasound treatment.

### Effect of Ultrasound on tPA Activity

The fibrinolytic activity of tPA was tested indirectly using the chromogenic substrate S‐2251 according to a previous report.^[^ ^49^ ^]^ Native tPA was added to a 96‐well plate containing buffer (0.1 M trihydrochloric acid) and S‐2251 (3.5 mM) along with fresh rabbit plasma (centrifuged at 5000 rpm for 10 min) at 37 °C. The samples were subjected to ultrasonication at the bottom of the well plate with sound intensities ranging from 0.5 to 2.0 W cm^−^^2^. The absorbance at 405 nm was measured at 2 min intervals for 40 min by a microplate reader. Fibrinolytic activity was calculated using the change in absorbance at 405 nm.

### In Vitro Thrombolysis Enhanced by Low‐Intensity Ultrasound

The clot model was generated as previously described. The following liquids were injected with a 50 µL syringe: 100 µL of normal saline solution and 100 µL of tPA solution (20 µg mL^−1^). In the thrombolysis experiments, the clot model was fixed at the bottom of a beaker filled with water, and the ultrasound probe immersed in water was placed above the clot. Ultrasound treatment with different sound intensity (0.5, 1, 1.5, 2, and 2.5 W cm^−^^2^) was applied for 1 h, and the distances between the ultrasound probes (d = 1, 2, 3, 4, and 5 cm) and the clots were investigated. The length of the clot was measured every 20 min by ultrasound imaging. (frequency: 12.5–23.0 MHz; depth: 2.0 cm; gain: 50; frequency rate: 12; dynamic range: 120, L20‐5U; Mindray Resona9 Pro, Mindray, China).

For the in vitro thrombolysis experiments in which pork was the medium, pork with a thickness of ≈2 cm was placed between the ultrasound probe and the clot. The thrombolytic efficiency was investigated with different ultrasound intensities (0.5, 1, 1.5 and 2 W cm^−2^), while the surface temperature of the pork was measured by an infrared camera.

### In Vitro Thrombolysis under TFV‐MF Combined with Low‐Intensity Ultrasound Treatment

The treatments were randomly divided into the following groups: NS group, NS+US group, tPA group, tPA+US group, tPA+TFR group, tPA+TFR+US group, tPA+ TFV group, and tPA +TFV+US group. After 100 µL of normal saline was injected, the following fluids were added: 100 µL of saline, a mixture of 50 µL of saline solution and 50 µL of tPA solution (100 µg mL^−1^), and a mixture of 50 µL of nanorobot dispersion (5 mg mL^−1^) and 50 µL of tPA solution (100 µg mL^−1^). The ultrasound power intensity was 0.5 W cm^−^^2^, and the rotation frequency was 2 Hz. All groups were treated for 1 h. Changes in clot length and area were recorded by operating microscopy and ultrasound imaging.

### In Vivo Thrombolysis

All procedures involving animals were approved by the Institutional Animal Care and Use Committee of Shanghai Sixth People's Hospital Affiliated to Shanghai Jiao Tong University School of Medicine (Animal Welfare Ethics acceptance number No. DWLL2024‐0665). Male SD rats (weighing 300 to 350 g) were anesthetized by intraperitoneal injection of 1% sodium pentobarbital and fixed in a supine position. A surgical incision was made in the rat's thigh area to expose the femoral vein vessels. The femoral vein vessel was wrapped with a piece of filter paper soaked with 10 wt.% FeCl_3_ (width of 2 mm) to form a thrombus. After ≈3 min, the filter paper was removed, and the vessel was washed with saline. The rat was placed on a 3‐DOF operating table platform with magnetic actuation system placed underneath. The action center of the magnetic actuation system was located on the femoral vein of the mouse. The low‐intensity ultrasound device was placed above the femoral vein thrombus site of rat with ultrasonic coupling agent.

Rats were treated with normal saline, native tPA, tPA+US or tPA+US+TFV. First, sodium heparin solution (60 U kg^−1^) was injected into the rats via the tail vein. Then, the rats in the tPA+US+TFV group were injected with 100 µL of magnetic colloid mixed with tPA solution (100 µg mL^−1^, equivalent to the amount of tPA in the tPA or tPA+US group) via the tail vein. The colloids were captured at the thrombus site. Then, TFV‐MF (2 Hz) was applied to form a collective and modulate the locomotion of colloids for thrombolysis. US (0.5 W cm^−^^2^) was also applied at the thrombus site simultaneously. The rats in the tPA and tPA+US groups were injected with 100 µL of tPA solution (100 µg mL^−1^) via the tail vein. The rats in the tPA+US group were treated with ultrasound (0.5 W cm^−^^2^) at the thrombus immediately after the tPA solution (100 µg mL^−1^) was injected. Femoral vein blood flow was monitored and recorded using a PeriCam PSI HR (Perimed AB, Sweden). The site of thrombosis was delineated as a region of interest (ROI). The perfusion in this area before thrombosis was defined as F1, the perfusion after thrombosis as F2, and the perfusion after different treatments in each group as F3. After 3 h of treatment, the relative perfusion recovery rate of the different treatment groups was calculated as follows:

(2) $$\mathrm{Relative}\;\mathrm{perfusion}\;=\frac{F3-F2}{F1-F2}$$

The femoral vein vascular status was also recorded with an operating microscope. At the end of the treatment, the vascular tissue at the thrombosis site was removed for histological analysis. ImageJ software was used to process and analyze the H&E‐stained sections. The ratio of the vascular occlusion region to the whole vessel was measured to determine the efficiency of thrombolysis.

### Cytocompatibility and Biocompatibility Assessment

The cytotoxicity of magnetic colloids was determined by a cell counting kit‐8 (CCK‐8) assay (Dojindo, Japan). Human umbilical vein endothelial cells (HUVECs) were cocultured with magnetic colloid at concentrations of 0, 25, 50, 100, 200, 400, and 800 µg mL^−1^. The change in absorbance at 450 nm was measured by a microplate reader.

The SD rats were intravenously injected with 100 µL of magnetic colloidal solution (5 mg mL^−1^). After 1 day and 7 days of feeding, the rats were euthanized, and the main organs were harvested for histological analysis. Moreover, fresh blood and plasma were collected for blood tests and biochemistry tests, respectively. SD rats in the control group were intravenously injected with 100 µL of normal saline solution (n = 3 for each group).

At the end of the low‐intensity ultrsound treatment, the vascular tissue at the thrombosis site was removed for histological analysis.

The hemocompatibility of the magnetic colloids was verified by a hemolysis assay. Fresh rabbit blood was obtained with an EDTA anticoagulation tube and then centrifuged at 3000×g at room temperature for 20 min. After washing three times with PBS, red blood cells (RBCs) were obtained from the precipitate. Colloids of different concentrations were incubated with RBCs for 3 h at room temperature. Erythrocytes incubated with PBS and DI water were used as negative and positive controls, respectively. After centrifugal ultrafiltration (10000×g), the absorbance of the supernatant at 540 nm was measured.

### Statistical Analysis

The data were expressed as the means ± SD. Inter‐ and intragroup comparisons and analyses in each experiment were performed by unpaired Student's t test and one‐way analysis of variance (ANOVA) using SPSS software. *p < 0.05, **p < 0.01 and ***p < 0.001 indicate significant differences.

## Conflict of Interest

The authors declare no conflict of interest.

## Author Contributions

L.W. and J.J.W. conceived the study, designed the experiments, and wrote the manuscript. L.W. and J.J.W. performed the experiments and analyzed data. W.Z. and T.J.Z helped the animal experiments. Q.L and Y.P. L helped the ultrasound imaging experiments. Y.H. L. gave suggestions on the animal experiments. L.W. and J.J.W checked the accuracy of the experimental results. L.Z and Y.Z. edited the paper. L.W and Y.Z supervised the project and all authors discussed the results and commented on the manuscript.

## Supporting information



Supporting Information

Supplemental Video 1

Supplemental Video 2

Supplemental Video 3

Supplemental Video 4

Supplemental Video 5

Supplemental Video 6

Supplemental Video 7

## Data Availability

The data that support the findings of this study are available in the supplementary material of this article.

## References

[1] D. A. D. S. Bruce , C. V. Campbell , M. R. Macleod , S. B. Coutts , L. H. Schwamm , S. M. Davis , G. A. Donnan , Nat. Rev. Dis. Primers. 2019, 5, 71. 31601801 10.1038/s41572-019-0118-8

[2] C. W. Tsao , A. W. Aday , Z. I. Almarzooq , A. Alonso , A. Z. Beaton , M. S. Bittencourt , A. K. Boehme , A. E. Buxton , A. P. Carson , Y. Commodore‐Mensah , M. S. V. Elkind , K. R. Evenson , C. Eze‐Nliam , J. F. Ferguson , G. Generoso , J. E. Ho , R. Kalani , S. S. Khan , B. M. Kissela , K. L. Knutson , D. A. Levine , T. T. Lewis , J. Liu , M. S. Loop , J. Ma , M. E. Mussolino , S. D. Navaneethan , A. M. Perak , R. Poudel , M. Rezk‐Hanna , et al., Circulation. 2022, 145, e153. 35078371 10.1161/CIR.0000000000001052

[3] T. Wang , H. Ugurlu , Y. Yan , M. Li , M. Li , A.‐M. Wild , E. Yildiz , M. Schneider , D. Sheehan , W. Hu , M. Sitti , Nat. Commun. 2022, 13, 4465. 35915075 10.1038/s41467-022-32059-9 PMC9343456

[4] M. O. McCarron , J. A. R. Nicoll , Lancet Neurol. 2004, 3, 484. 15261609 10.1016/S1474-4422(04)00825-7

[5] E. J. Tilling , S. E. Tawil , K. W. Muir , Stroke. 2019, 50, 344. 30626290 10.1161/STROKEAHA.118.022606

[6] M. S. Phipps , C. A. Cronin , BMJ. 2020, 368, l6983. 32054610 10.1136/bmj.l6983

[7] B. J. Nelson , S. Pané , Science. 2023, 382, 1120. 38060660 10.1126/science.adh3073

[8] N. Li , P. Fei , C. Tous , M. Rezaei Adariani , M.‐L. Hautot , I. Ouedraogo , A. Hadjadj , I. P. Dimov , Q. Zhang , S. Lessard , Z. Nosrati , C. N. Ng , K. Saatchi , U. O. Häfeli , C. Tremblay , S. Kadoury , A. Tang , S. Martel , G. Soulez , Sci. Robot. 2024, 9, eadh8702. 38354257 10.1126/scirobotics.adh8702

[9] X. Liu , L. Wang , Y. Xiang , F. Liao , N. Li , J. Li , J. Wang , Q. Wu , C. Zhou , Y. Yang , Y. Kou , Y. Yang , H. Tang , N. Zhou , C. Wan , Z. Yin , G.‐Z. Yang , G. Tao , J. Zang , Sci. Robot. 2024, 9, eadh2479. 38381840 10.1126/scirobotics.adh2479

[10] B. Sun , S. Kjelleberg , J. J. Y. Sung , L. Zhang , Nat. Rev. Bioeng. 2024, 2, 367.

[11] S. A. Abbasi , A. Ahmed , S. Noh , N. L. Gharamaleki , S. Kim , A. M. M. B. Chowdhury , J.‐y. Kim , S. Pané , B. J. Nelson , H. Choi , Nat. Mach. Intell. 2024, 6, 92.

[12] M. E. Tiryaki , Y. G. Elmacıoğlu , M. Sitti , Sci. Adv. 2023, 9, eadg6438. 37126547 10.1126/sciadv.adg6438 PMC10132757

[13] D. Jin , Q. Wang , K. F. Chan , N. Xia , H. Yang , Q. Wang , S. C. H. Yu , L. Zhang , Sci. Adv. 2023, 9, eadf9278. 37172097 10.1126/sciadv.adf9278 PMC10181194

[14] H. Zhou , C. C. Mayorga‐Martinez , S. Pané , L. Zhang , M. Pumera , Chem. Rev. 2021, 121, 4999. 33787235 10.1021/acs.chemrev.0c01234 PMC8154323

[15] Q. Wang , Q. Wang , Z. Ning , K. F. Chan , J. Jiang , Y. Wang , L. Su , S. Jiang , B. Wang , B. Y. M. Ip , H. Ko , T. W. H. Leung , P. W. Y. Chiu , S. C. H. Yu , L. Zhang , Sci. Robot. 2024, 9, eadh1978. 38381838 10.1126/scirobotics.adh1978

[16] H. Zhang , Z. Li , C. Gao , X. Fan , Y. Pang , T. Li , Z. Wu , H. Xie , Q. He , Sci. Robot. 2021, 6, eaaz9519. 34043546 10.1126/scirobotics.aaz9519

[17] B. Wang , K. F. Chan , K. Yuan , Q. Wang , X. Xia , L. Yang , H. Ko , Y.‐X. J. Wang , J. J. Y. Sung , P. W. Y. Chiu , L. Zhang , Sci. Robot. 2021, 6, eabd2813. 34043547 10.1126/scirobotics.abd2813

[18] P. E. Dupont , B. J. Nelson , M. Goldfarb , B. Hannaford , A. Menciassi , M. K. O'Malley , N. Simaan , P. Valdastri , G.‐Z. Yang , Sci. Robot. 2021, 6, eabi8017. 34757801 10.1126/scirobotics.abi8017 PMC8890492

[19] P. Wrede , O. Degtyaruk , S. K. Kalva , X. L. Deán‐Ben , U. Bozuyuk , A. Aghakhani , B. Akolpoglu , M. Sitti , D. Razansky , Sci. Adv. 2022, 8, eabm9132. 35544570 10.1126/sciadv.abm9132 PMC9094653

[20] J. Law , X. Wang , M. Luo , L. Xin , X. Du , W. Dou , T. Wang , G. Shan , Y. Wang , P. Song , X. Huang , J. Yu , Y. Sun , Sci. Adv. 2022, 8, eabm5752. 35857830 10.1126/sciadv.abm5752 PMC9299543

[21] H. Gu , E. Hanedan , Q. Boehler , T.‐Y. Huang , A. J. T. M. Mathijssen , B. J. Nelson , Nat. Mach. Intell. 2022, 4, 678.

[22] L. Wang , W. Zou , J. Shen , S. Yang , J. Wu , T. Ying , X. Cai , L. Zhang , J. Wu , Y. Zheng , Adv. Healthcare Mater. 2024, 13, 2303361. 10.1002/adhm.202303361 38115718

[23] M. Xie , W. Zhang , C. Fan , C. Wu , Q. Feng , J. Wu , Y. Li , R. Gao , Z. Li , Q. Wang , Y. Cheng , B. He , Adv. Mater. 2020, 32, 2000366. 10.1002/adma.202000366 32430939

[24] S. Wang , X. Guo , W. Xiu , Y. Liu , L. Ren , H. Xiao , F. Yang , Y. Gao , C. Xu , L. Wang , Sci. Adv. 2020, 6, eaaz8204. 32832678 10.1126/sciadv.aaz8204 PMC7439573

[25] X. Tang , L. Manamanchaiyaporn , Q. Zhou , C. Huang , L. Li , Z. Li , L. Wang , J. Wang , L. Ren , T. Xu , X. Yan , Y. Zheng , Small. 2022, 18, 2202848. 10.1002/smll.202202848 35905497

[26] M. Yang , Y. Zhang , F. Mou , C. Cao , L. Yu , Z. Li , J. Guan , Sci. Adv. 2023, 9, eadk7251. 38019908 10.1126/sciadv.adk7251 PMC10686566

[27] B. Wang , Q. Wang , K. F. Chan , Z. Ning , Q. Wang , F. Ji , H. Yang , S. Jiang , Z. Zhang , B. Y. M. Ip , H. Ko , J. P. W. Chung , M. Qiu , J. Han , P. W. Y. Chiu , J. J. Y. Sung , S. Du , T. W. H. Leung , S. C. H. Yu , L. Zhang , Sci. Adv. 2024, 10, eadk8970. 38295172 10.1126/sciadv.adk8970 PMC10830105

[28] L. Wang , J. Wang , J. Hao , Z. Dong , J. Wu , G. Shen , T. Ying , L. Feng , X. Cai , Z. Liu , Y. Zheng , Adv. Mater. 2021, 33, 2105351. 10.1002/adma.202105351 34647345

[29] Q. Wang , D. Jin , B. Wang , N. Xia , H. Ko , B. Y. M. Ip , T. W. H. Leung , S. C. H. Yu , L. Zhang , IEEE‐ASME Trans. Mechatron. 2022, 27, 2267.

[30] U. Bozuyuk , H. Ozturk , M. Sitti , Adv. Intell. Syst. 2023, 5, 2300099.

[31] Z. Wu , Y. Zhang , N. Ai , H. Chen , W. Ge , Q. Xu , Adv. Intell. Syst. 2022, 4, 2100266.

[32] J. Law , H. Chen , Y. Wang , J. Yu , Y. Sun , Sci. Adv. 2022, 8, eade3161. 36399567 10.1126/sciadv.ade3161 PMC9674281

[33] H. Xu , M. Medina‐Sánchez , M. F. Maitz , C. Werner , O. G. Schmidt , ACS Nano. 2020, 14, 2982. 32096976 10.1021/acsnano.9b07851

[34] Y. Alapan , U. Bozuyuk , P. Erkoc , A. C. Karacakol , M. Sitti , Sci. Robot. 2020, 5, eaba5726. 33022624 10.1126/scirobotics.aba5726

[35] Q. Wang , X. Du , D. Jin , L. Zhang , ACS Nano. 2022, 16, 604. 34985859 10.1021/acsnano.1c07830

[36] Q. Wang , K. F. Chan , K. Schweizer , X. Du , D. Jin , S. C. H. Yu , B. J. Nelson , L. Zhang , Sci. Adv. 2021, 7, eabe5914. 33637532 10.1126/sciadv.abe5914 PMC7909881

[37] T. Li , S. Yu , B. Sun , Y. Li , X. Wang , Y. Pan , C. Song , Y. Ren , Z. Zhang , K. T. V. Grattan , Z. Wu , J. Zhao , Sci. Adv. 2023, 9, eadg4501. 37146139 10.1126/sciadv.adg4501 PMC10162671

[38] L. Wang , H. Gao , H. Sun , Y. Ji , L. Song , L. Jia , C. Wang , C. Li , D. Zhang , Y. Xu , H. Chen , L. Feng , Research. 2023, 6, 0088. 36996337 10.34133/research.0088 PMC10042322

[39] M. Sun , B. Hao , S. Yang , X. Wang , C. Majidi , L. Zhang , Nat. Commun. 2022, 13, 7919. 36564394 10.1038/s41467-022-35646-y PMC9789085

[40] T. Gwisai , N. Mirkhani , M. G. Christiansen , T. T. Nguyen , V. Ling , S. Schuerle , Sci. Robot. 2022, 7, eabo0665. 36288270 10.1126/scirobotics.abo0665

[41] N. Mirkhani , M. G. Christiansen , T. Gwisai , S. Menghini , S. Schuerle , Nat. Commun. 2024, 15, 2160. 38461256 10.1038/s41467-024-46407-4 PMC10924878

[42] Z. Meng , Y. Zhang , E. Shen , W. Li , Y. Wang , K. Sathiyamoorthy , W. Gao , M. C. Kolios , W. Bai , B. Hu , W. Wang , Y. Zheng , Adv. Sci. 2021, 8, 2004670. 10.1002/advs.202004670 PMC8261514 34258156

[43] Y. Zhong , Y. Zhang , J. Xu , J. Zhou , J. Liu , M. Ye , L. Zhang , B. Qiao , Z.‐g. Wang , H.‐t. Ran , D. Guo , ACS Nano. 2019, 13, 3387. 30855938 10.1021/acsnano.8b09277

[44] S. Guo , X. Guo , X. Wang , D. Zhou , X. Du , M. Han , Y. Zong , M. Wan , Ultrason. Sonochem. 2019, 54, 183. 30773494 10.1016/j.ultsonch.2019.02.001

[45] S. Guo , Z. Ya , P. Wu , L. Zhang , M. Wan , Ultrasound Med. Biol. 2022, 48, 1907. 35764456 10.1016/j.ultrasmedbio.2022.05.021

[46] Z. Ma , C. Bourquard , Q. Gao , S. Jiang , T. De Iure‐Grimmel , R. Huo , X. Li , Z. He , Z. Yang , G. Yang , Y. Wang , E. Lam , Z.‐h. Gao , O. Supponen , J. Li , Science. 2022, 377, 751. 35951702 10.1126/science.abn8699

[47] B. Zhang , H. Wu , H. Kim , P. J. Welch , A. Cornett , G. Stocker , R. G. Nogueira , J. Kim , G. Owens , P. A. Dayton , Z. Xu , C. Shi , X. Jiang , Research. 2023, 6, 0048. 37040522 10.34133/research.0048 PMC10078321

[48] H. Deng , X. Li , Q. Peng , X. Wang , J. Chen , Y. Li , Angew. Chem.‐Int. Edit. 2005, 44, 2782. 10.1002/anie.200462551 15798982

[49] H. Shimada , T. Mori , A. Takada , Y. Takada , Y. Noda , I. Takai , H. Kohda , T. Nishimura , J. Thromb. Haemost. 1981, 46, 507. 7197812

