# Supplementary material for: Cilia‐Mimic Locomotion of Magnetic Colloidal Collectives Enhanced by Low‐Intensity Ultrasound for Thrombolytic Drug Penetration
Source: Adv Sci (Weinh). 2024 Dec 27;12(7):2410351. doi: 10.1002/advs.202410351 (PMC11831500; doi:10.1002/advs.202410351)
Supplement: Supplementary file 1 — Supporting Information [file ADVS-12-2410351-s001.pdf]

ADVANCED SCIENCE
Open Access

Supporting Information

for *Adv. Sci.*, DOI 10.1002/advs.202410351

Cilia-Mimic Locomotion of Magnetic Colloidal Collectives Enhanced by Low-Intensity Ultrasound for Thrombolytic Drug Penetration

Jingjing Wu, Weijuan Zou, Qijie Lu, Tingjia Zheng, Yanping Li, Tao Ying, Yuehua Li, Yuanyi Zheng and Longchen Wang**

Supporting Information

Cilia-mimic locomotion of magnetic colloidal collectives enhanced by low-intensity ultrasound for thrombolytic drug penetration

Dr. J. Wu, Dr. W. Zou, Ms. T. Zheng, Dr. Q. Lu, Dr. Y.P. Li, Prof. T. Ying, Prof. Y. Zheng, Prof. L. Wang
Department of Ultrasound in Medicine, Shanghai Sixth People's Hospital Affiliated to Shanghai Jiao Tong University School of Medicine, No. 600, Yishan Road, Shanghai, 200233, P. R. China.
Shanghai Key Laboratory of Neuro-Ultrasound for Diagnosis and Treatment, Shanghai, P.R. China 200233
E-mail: zhengyuanyi@sjtu.edu.cn; wanglch09@sjtu.edu.cn

Prof. Y.H. Li
Department of Radiology, Shanghai Sixth People's Hospital Affiliated to Shanghai Jiao Tong University School of Medicine, No. 600, Yishan Road, Shanghai, P. R. China.

This PDF file includes:

Supplementary Materials

Figs. S1. to S33

Supplementary videos

Video S1. Cilia-mimic locomotion of magnetic colloidal collectives under TFV-MF from the side view.

Video S2. TFV-MF actuated magnetic colloids to form collectives and movement.

Video S3. Trajectory locomotion of magnetic colloidal collectives under TFV-MF.

Video S4. B-mode ultrasound imaging of magnetic colloids under TFR-MF and TFV-MF.

Video S5. Color doppler ultrasound imaging of magnetic colloids under TFR-MF and TFV-MF.

Video S6. Dye molecules diffusion enhanced by ultrasound.

Video S7. Locomotion of magnetic colloidal collectives in rat femoral vein in-vivo under TFV-MF.

SUPPLEMENTARY FIGURES

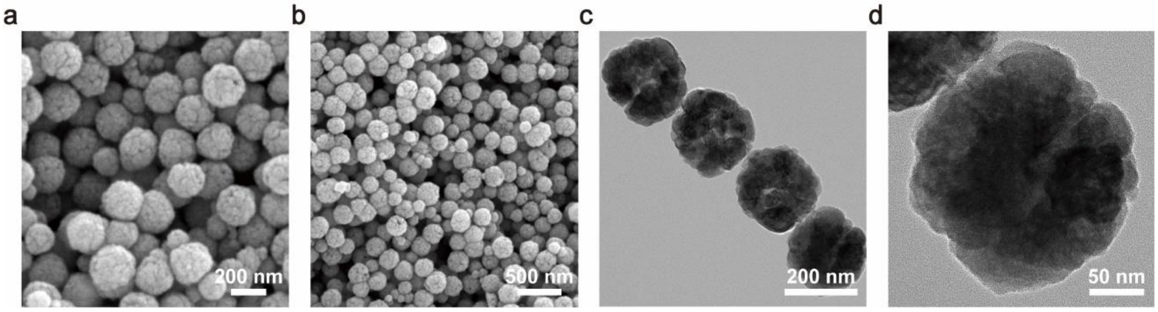

Figure S1. Characterization of the magnetic Fe_3O_4 colloids. SEM image (**a** and **b**) and TEM image (**c** and **d**).

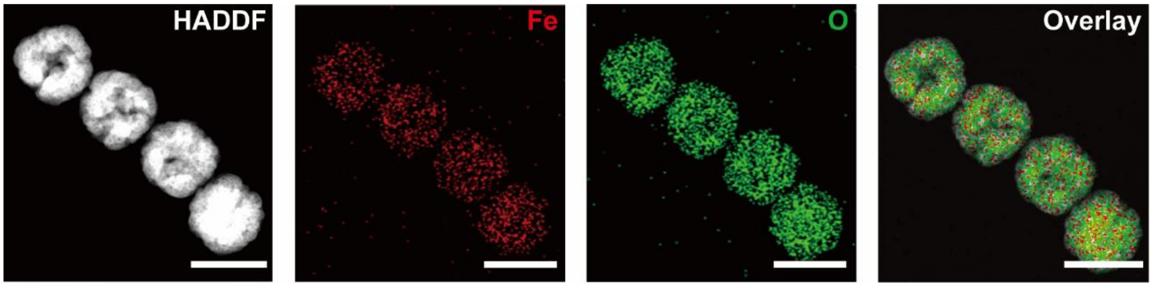

Figure S2. Element mapping of Fe and O from the magnetic Fe_3O_4 colloids. Scale bar, 200 nm. HAADF: high angle annular dark field.

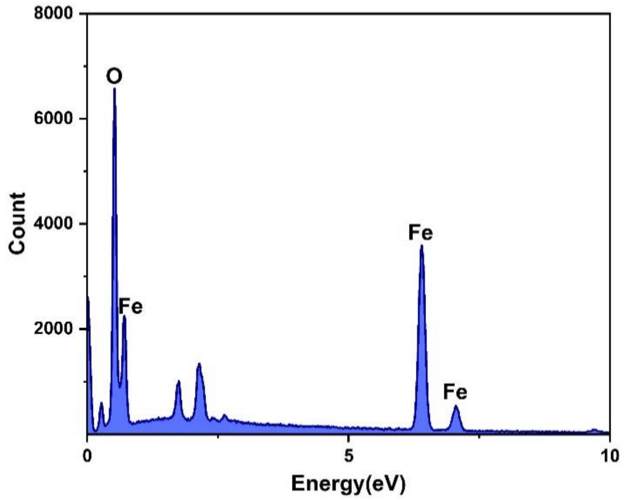

Figure S3 EDX analysis demonstrating the element of Fe and O from the magnetic Fe_3O_4 colloids.

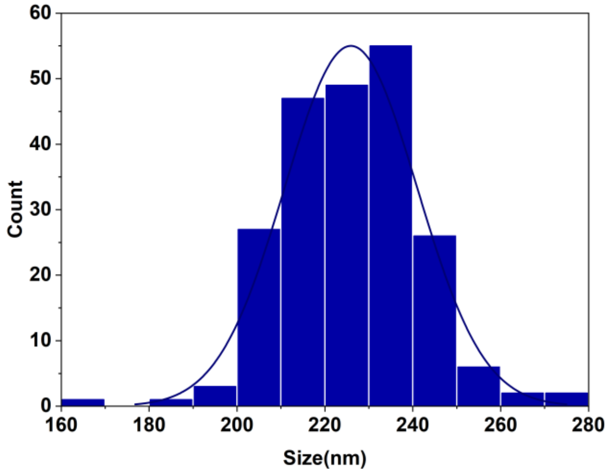

Figure S4 Size-distribution histogram of the magnetic Fe_3O_4 colloids.

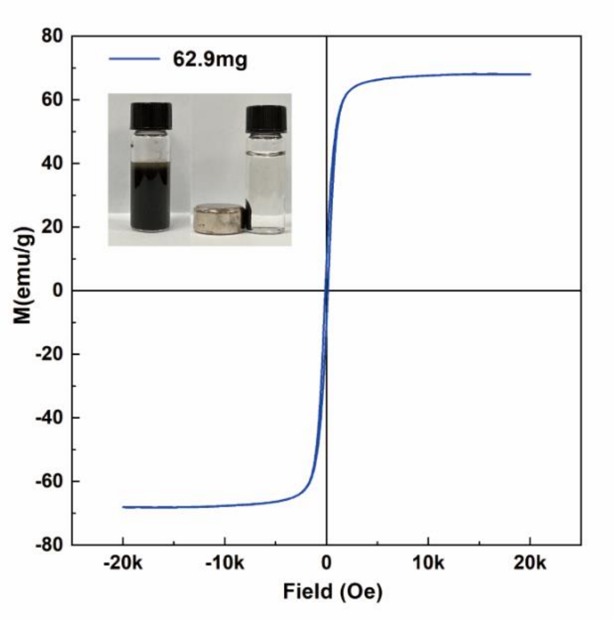

Figure S5 Static hysteresis loop of the magnetic colloids. The inset depicting the colloids separated rapidly from the dispersion solution by a magnet.

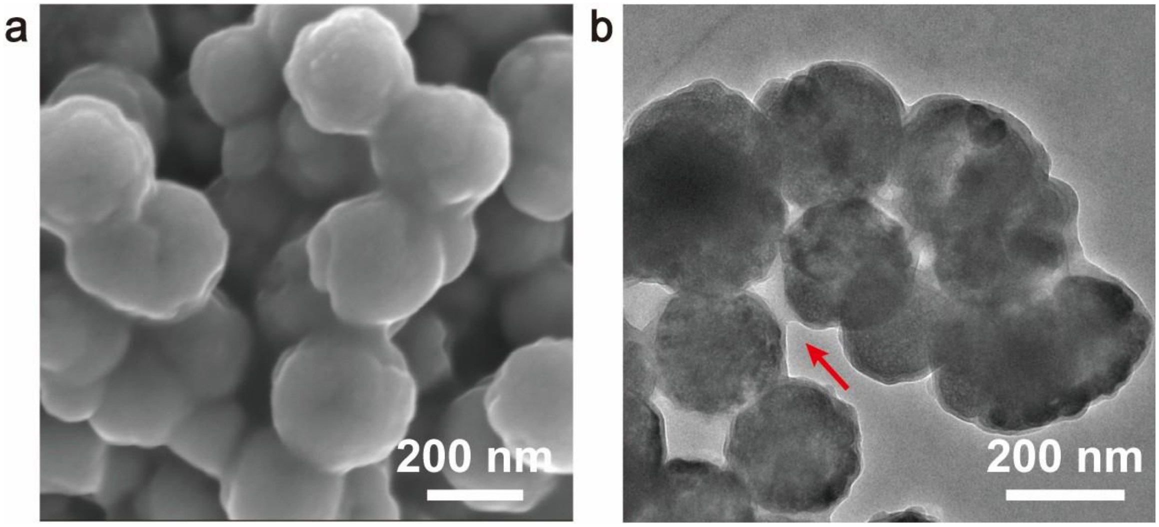

Figure S6. Characterization of the Fe_3O_4 colloids with tPA on the surface. (**a**)SEM and (**b**)TEM imaging. The red arrow showing the adsorbed substances on the magnetic Fe_3O_4 colloids surface.

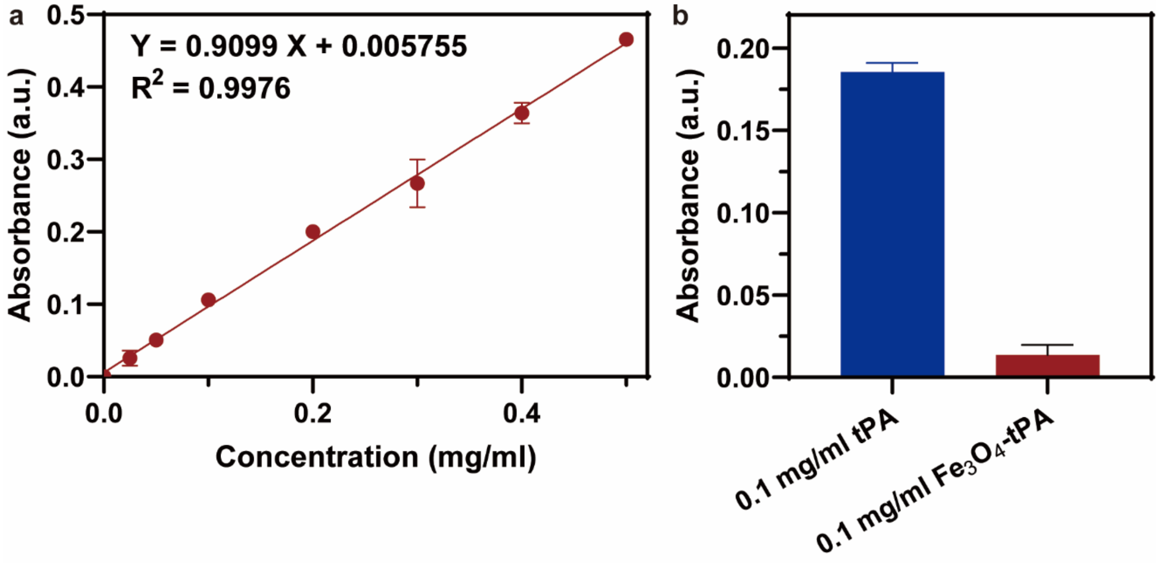

Figure S7. Calculation of loading dose of tPA after mixing with magnetic colloids. (**a**)The standard curve of absorption intensity for tPA as a function of its concentration. (**b**) Bar graph showing the absorbance of the tPA and Fe_3O_4-tPA with concentration of 0.1 mg/ml.

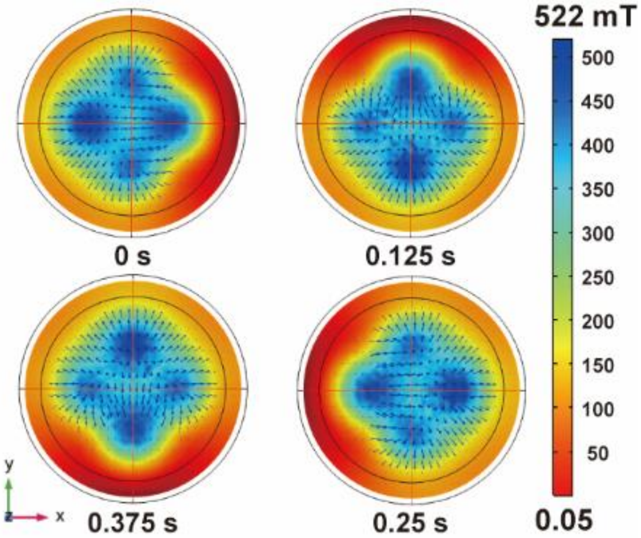

Figure S8. Simulation results showing the periodic change of magnetic field at a frequency of 2 Hz by COMSOL Multiphysics.

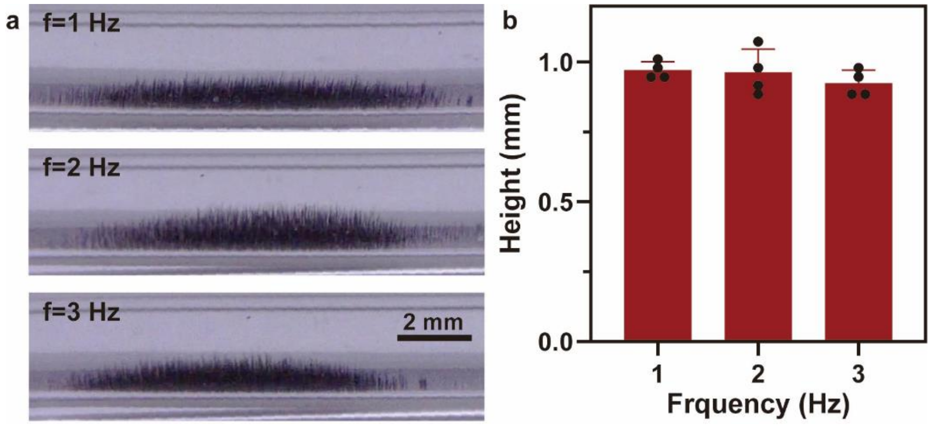

Figure S9. The height of cilia-mimic colloids at different frequencies from 1 Hz to 3 Hz.

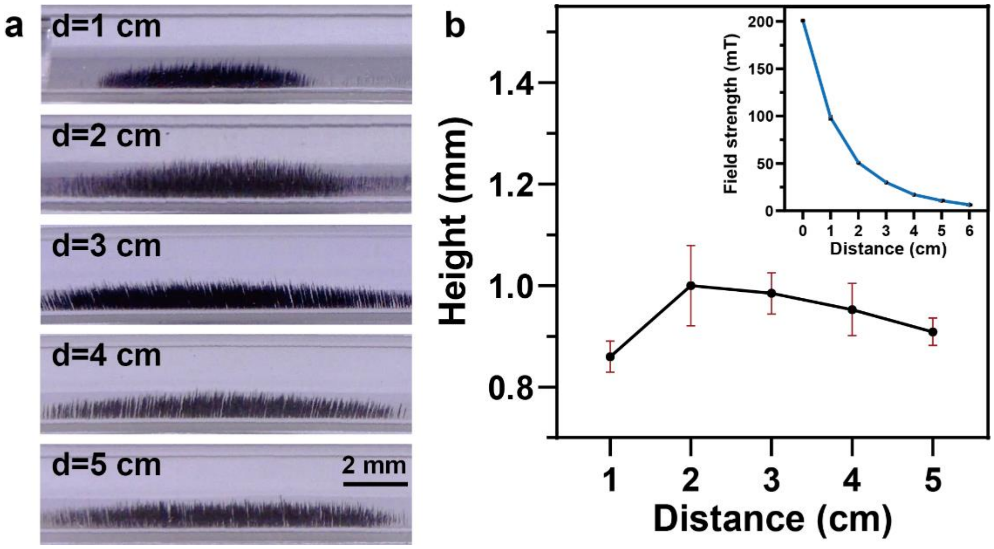

Figure S10. The height variation of cilia-mimic colloids with different distances separating from magnetic actuation system at the frequency of 2 Hz. The inset shows the strength of the magnetic field at different distances.

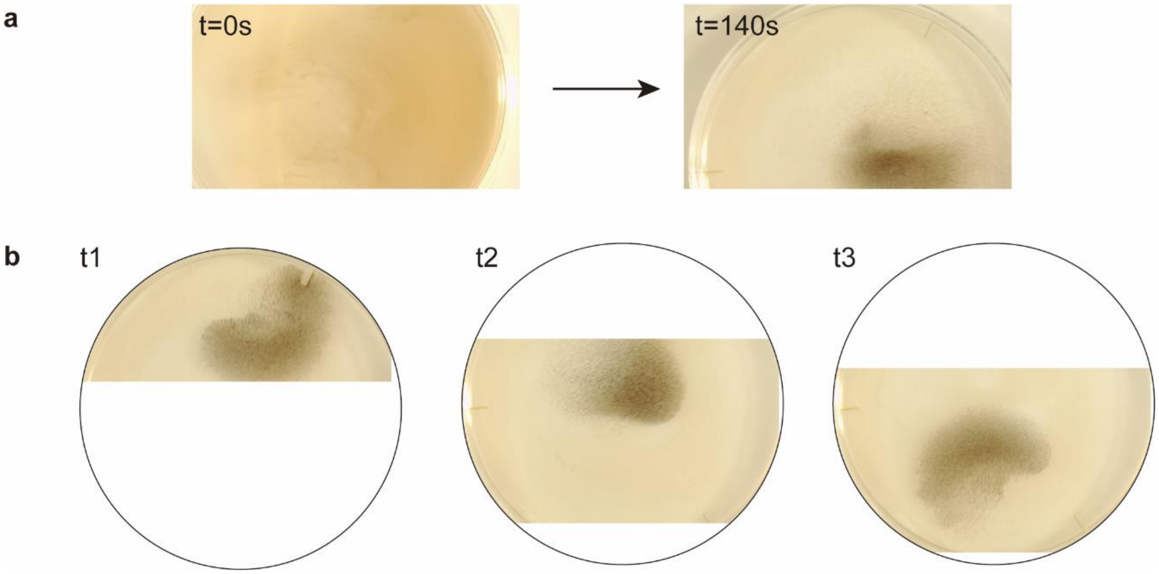

Figure S11. Magnetic actuated locomotion of colloids under TFV-MF. (A) TFV-MF actuated colloids to form collectives. (B) Magnetic actuated directional movement of colloidal collectives under TFV-MF.

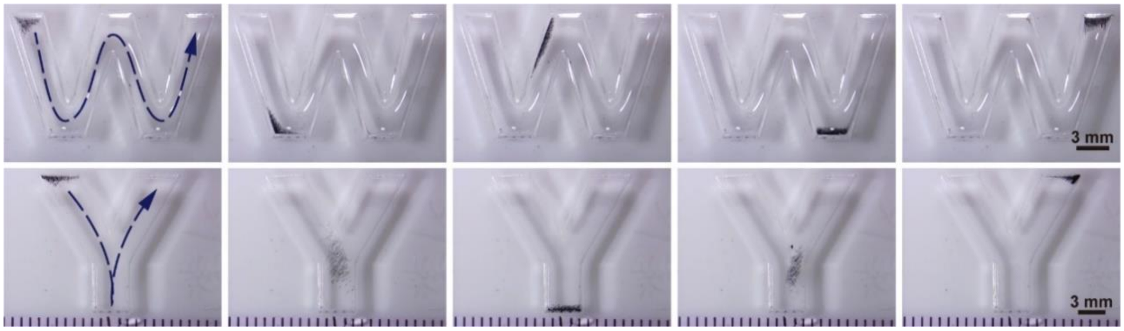

Figure S12 Snapshots showing the actuation of the magnetic colloids under TFV-MF in a “W”-shaped and a “Y”-shaped mold from the top view.

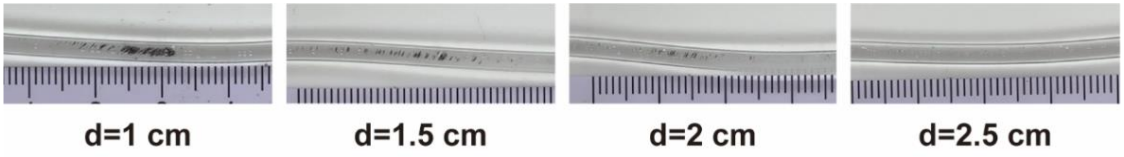

Figure S13 Representative optical images of magnetic colloids captured with distance variation.

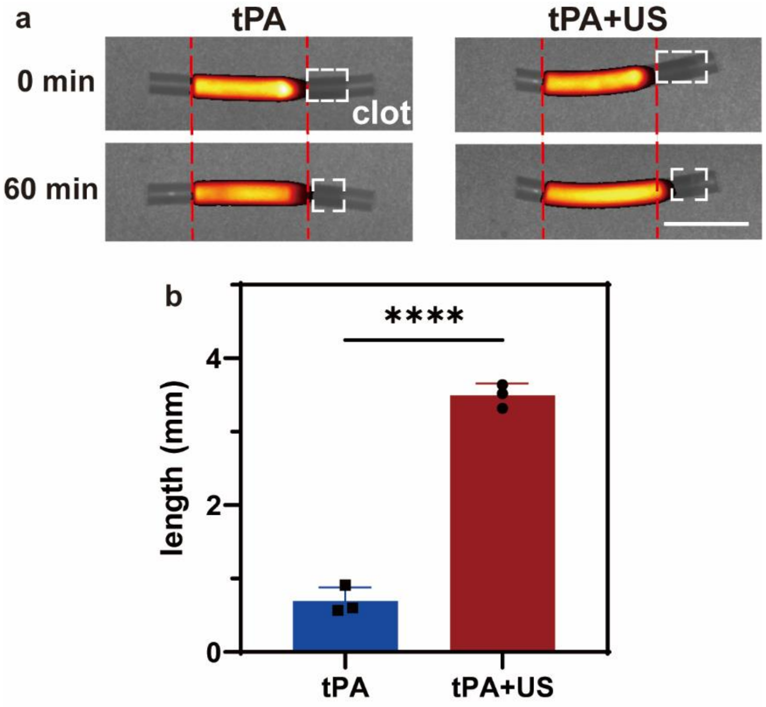

Figure S14. Ultrasound enhancing penetration the Cy 5.5 fluorescent dye into clots. (**A**) Representative images showing the penetration of Cy 5.5 mixed tPA into the interior of clots under ultrasound treatment. Scale bar, 1 cm. (**B**) Quantification of the length change of Cy 5.5 fluorescent dye under different treatments.

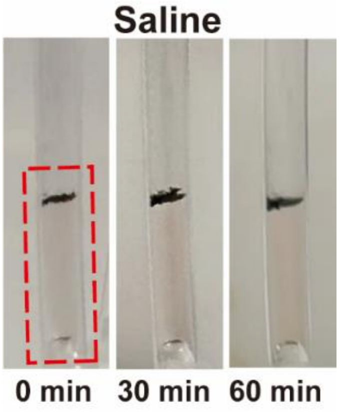

Figure S15. Penetration of magnetic colloids into transparent of clot treated by normal saline in 60 min. No obvious change of transparent clot was observed, which indicating the firm formation of transparent clot.

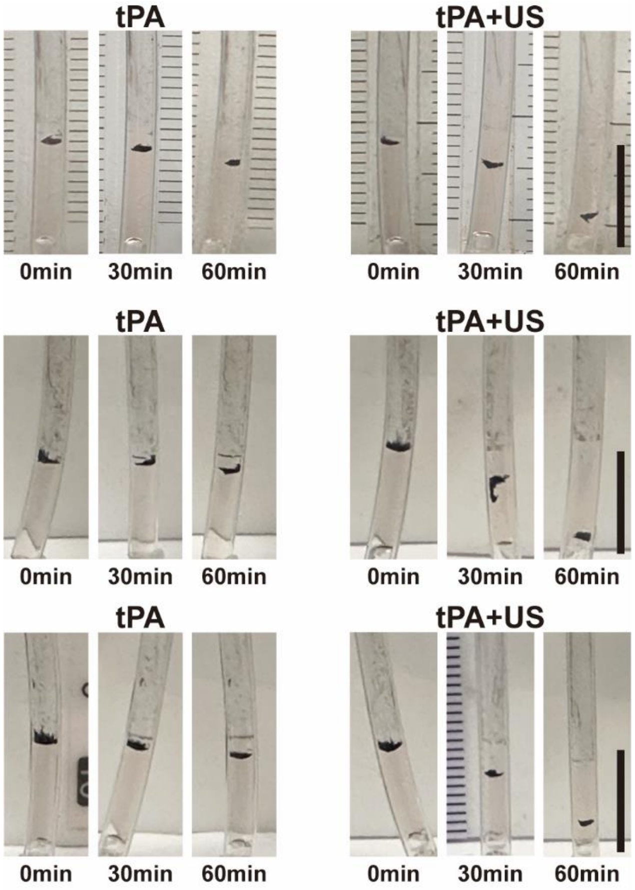

Figure S16. The process of magnetic colloids penetration into clots under different treatments in 60 min. (n=3). Scale Bar,1 cm.

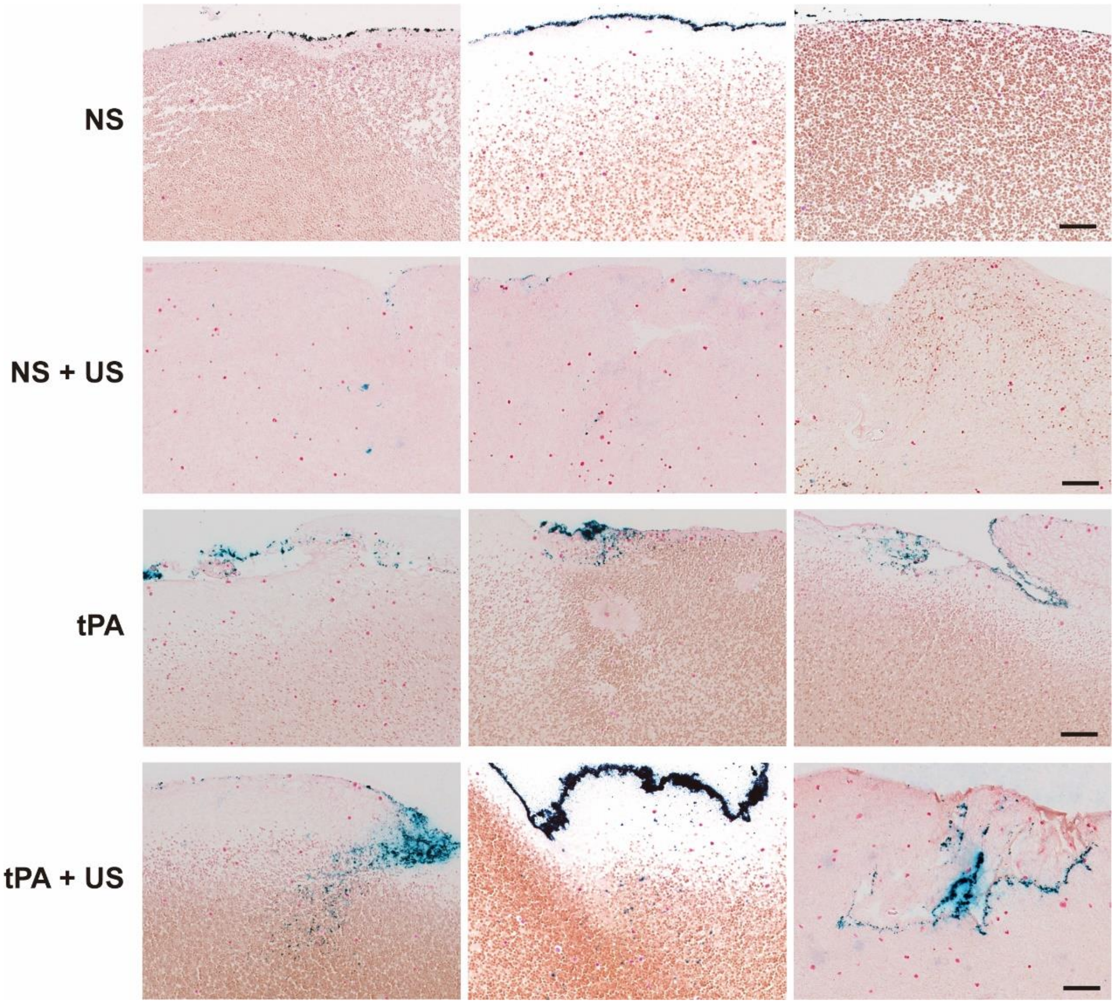

Figure S17. Prussian blue staining showing magnetic colloids in red clot slice under different treatments. Scale Bar, 50 μm.

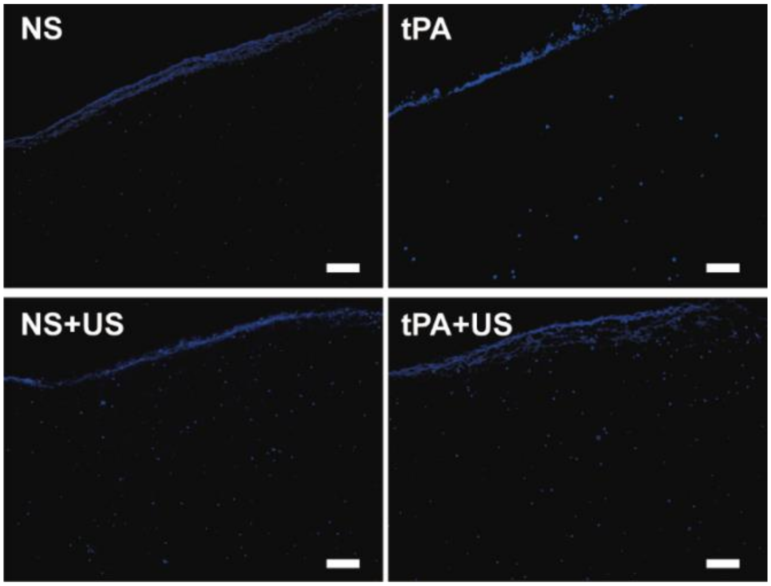

Figure S18. The fluorescent images showing the penetration of dppa stained colloids (blue) into the interior of red clots under different treatments. Scale Bar, 100 μm.

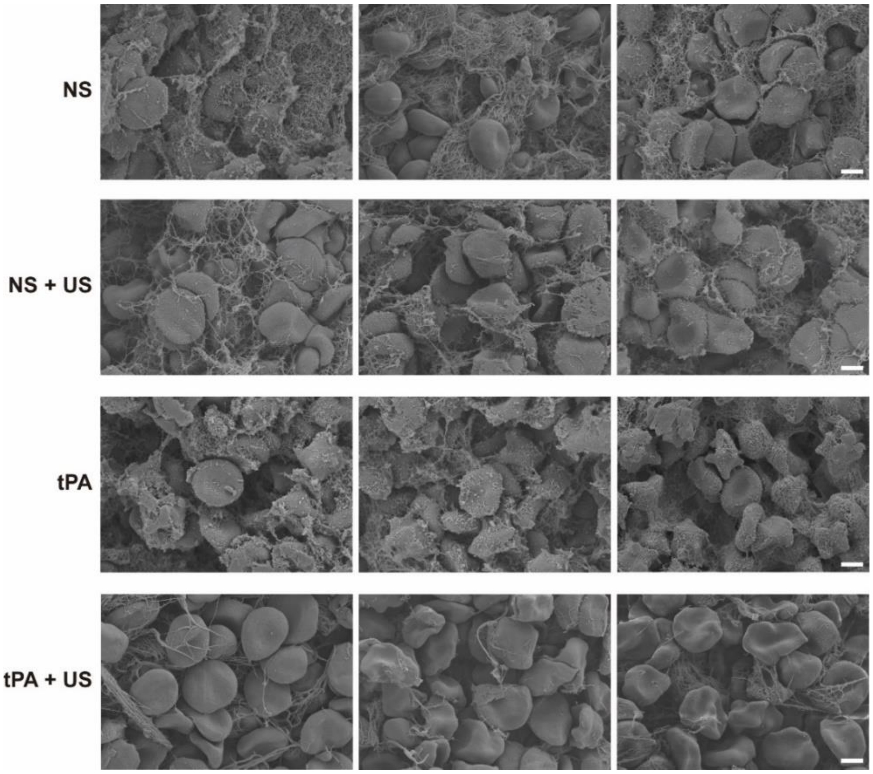

Figure S19. The SEM images showing the morphological structure change of red clots under different treatments. Scale Bar, 2 μm.

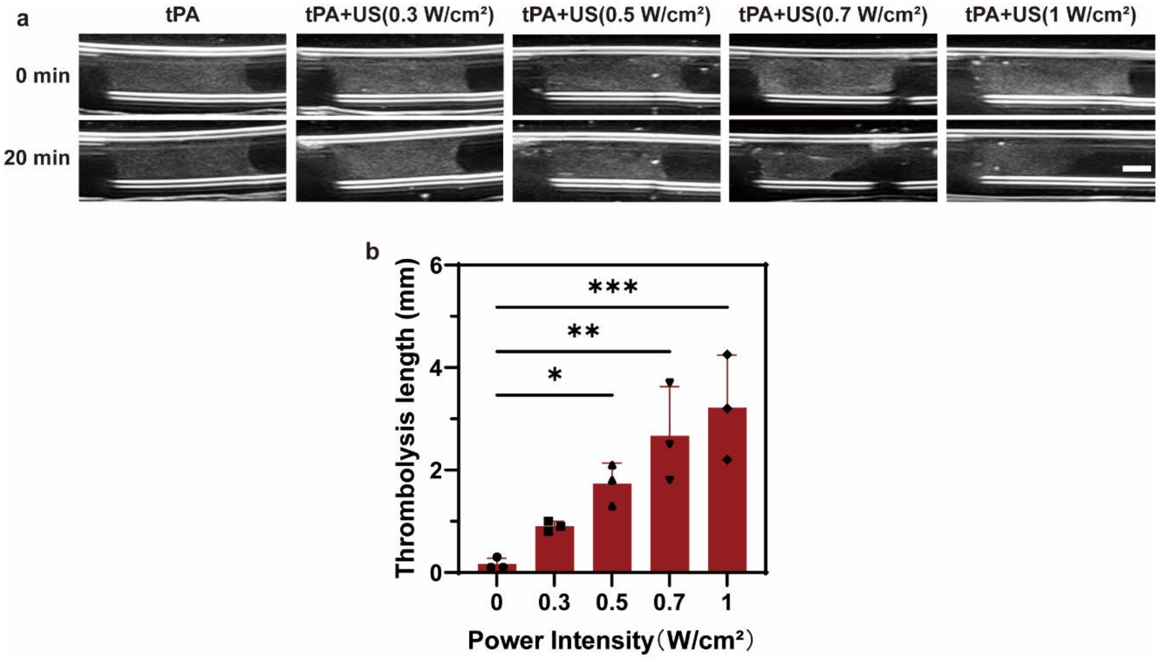

Figure S20. In vitro thrombolysis with different ultrasound power intensity. (a) Representative images of the thrombus length change in 20 minutes after treated by ultrasound with different sound intensity (0, 0.3, 0.5, 0.7, 1 W/cm^2, respectively). The ultrasound probe was deployed in tact with the thrombus directly. Scale Bars, 2 mm. **(b)** Quantitative analysis of thrombolysis length treated by ultrasound with different sound intensity.

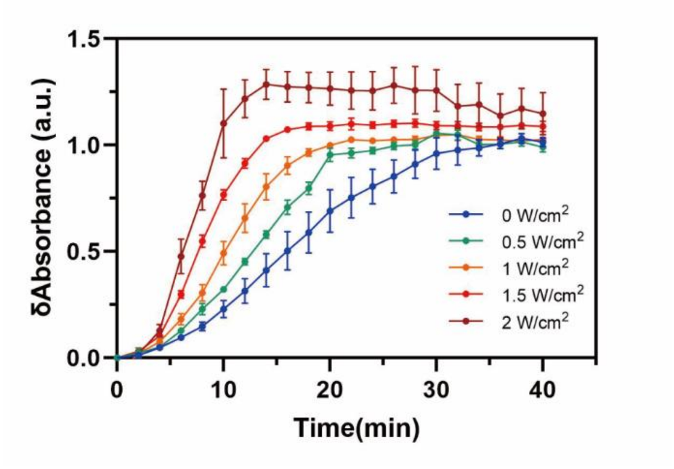

Figure S21. The change of absorbance of the reaction system at different time intervals at OD= 405 nm with native tPA, and ultrasound treatment with different sound intensity, respectively.

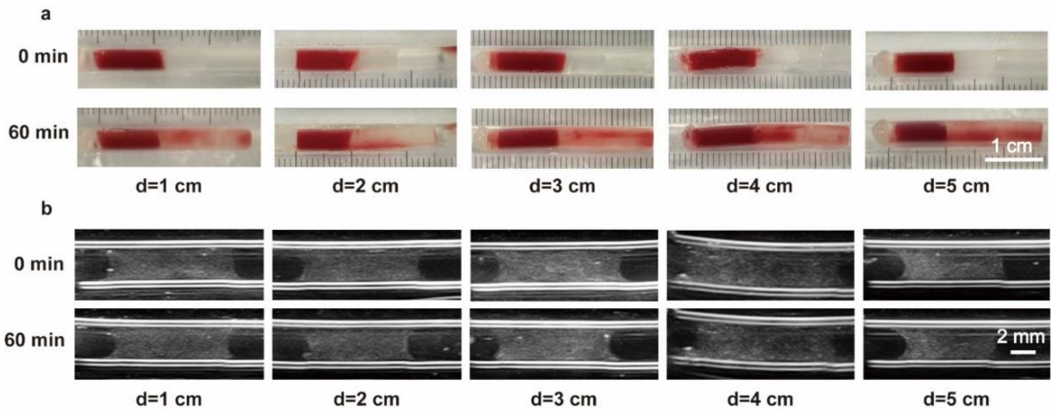

Figure S22. Snapshot (**a**) and ultrasound images (**b**) of the thrombus treated by ultrasound in 60 min with different distances between the probe and the clot. Water is used as medium.

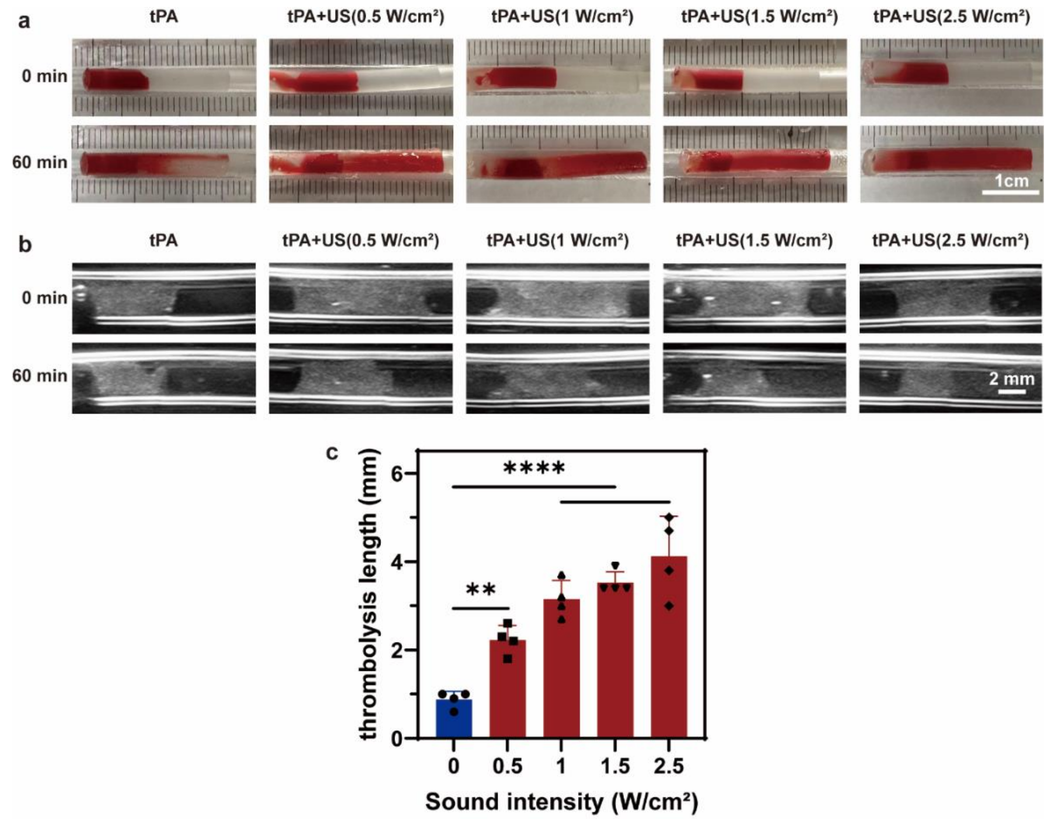

Figure S23. (**a**) Snapshot of the thrombolysis process treated by ultrasound with different sound intensity (0, 0.5, 1, 1.5, 2.5 W/cm^2, respectively) using pork as a medium. Scale bar, 1 cm. (**b**) Corresponding ultrasound images of the thrombus treated by ultrasound with different sound intensity. Scale bar, 2 mm. (**c**) Quantitative evaluation of thrombolysis length treated by ultrasound with different sound intensity (0, 0.5, 1, 1.5, 2.5 W/cm^2, respectively).

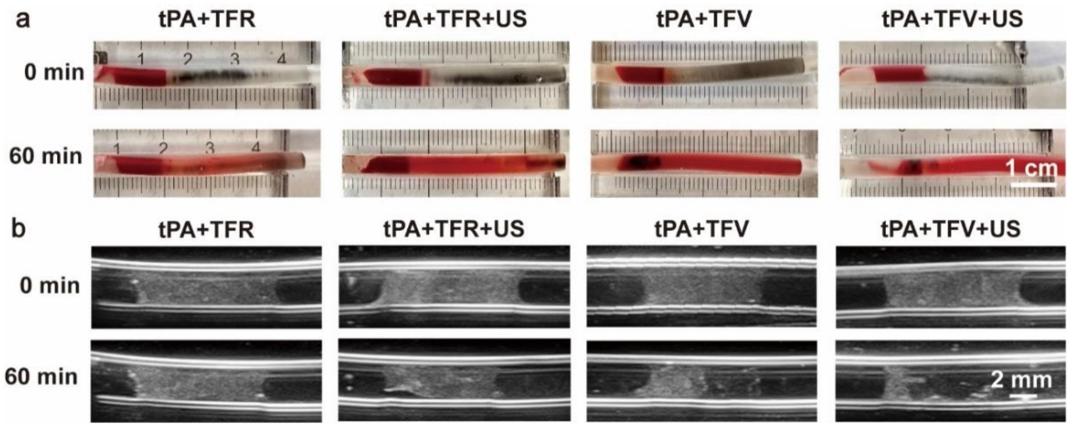

Figure S24. Snapshot (**a**) and ultrasound image (**b**) of the thrombus under different treatment in 60 min.

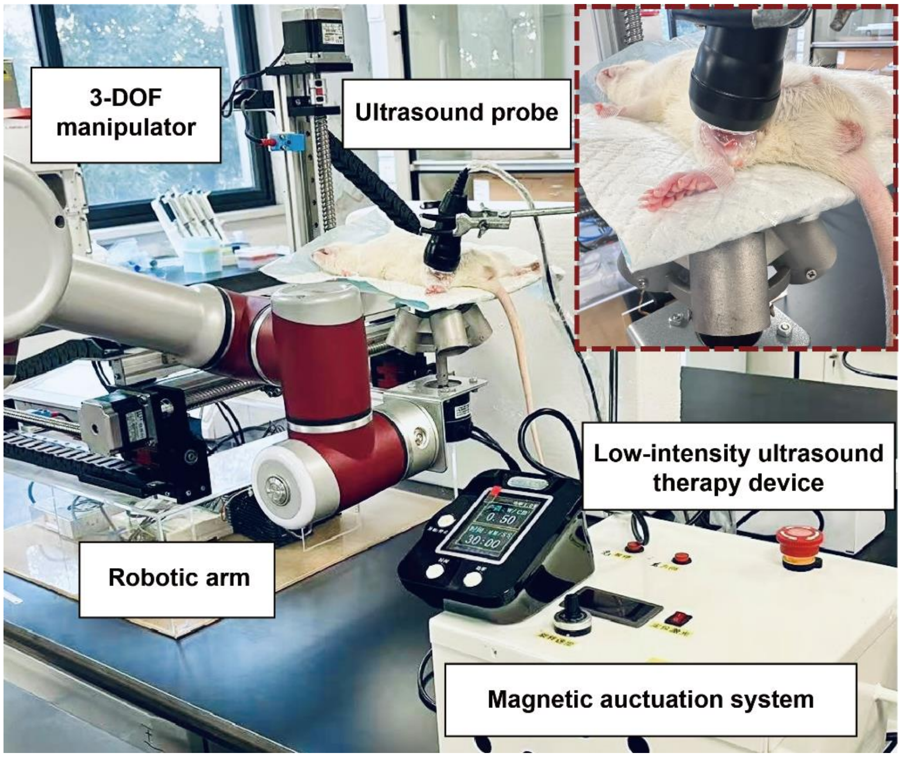

Figure S25. Magnetic actuation system and low-intensity ultrasound device for rat femoral venous thrombus therapy

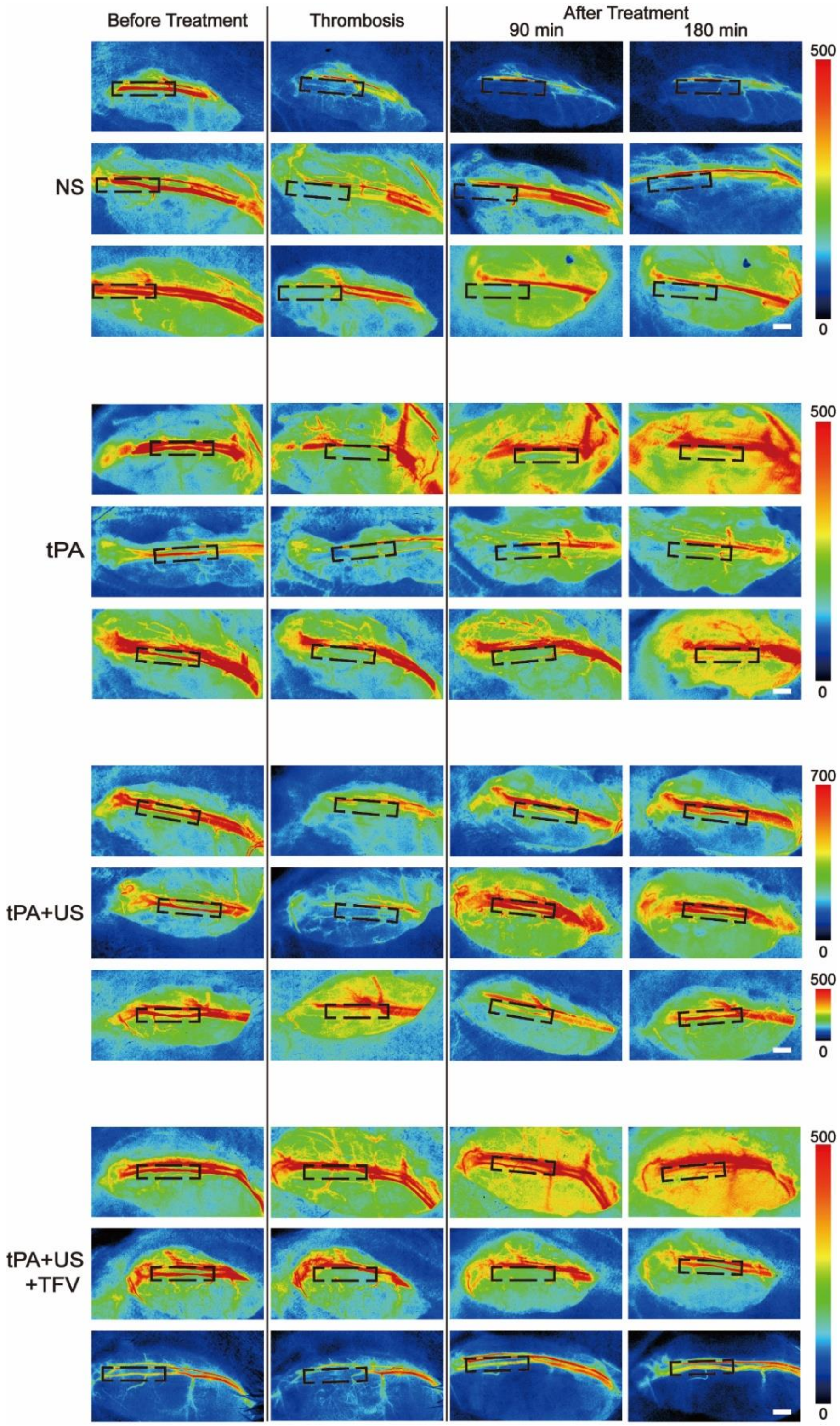

Figure S26. Pericam-monitored blood flow change in the femoral vein of the SD rats treated with NS, tPA, tPA+US and tPA+US+TFV in 180 min, respectively. Scale bar, 2 mm.

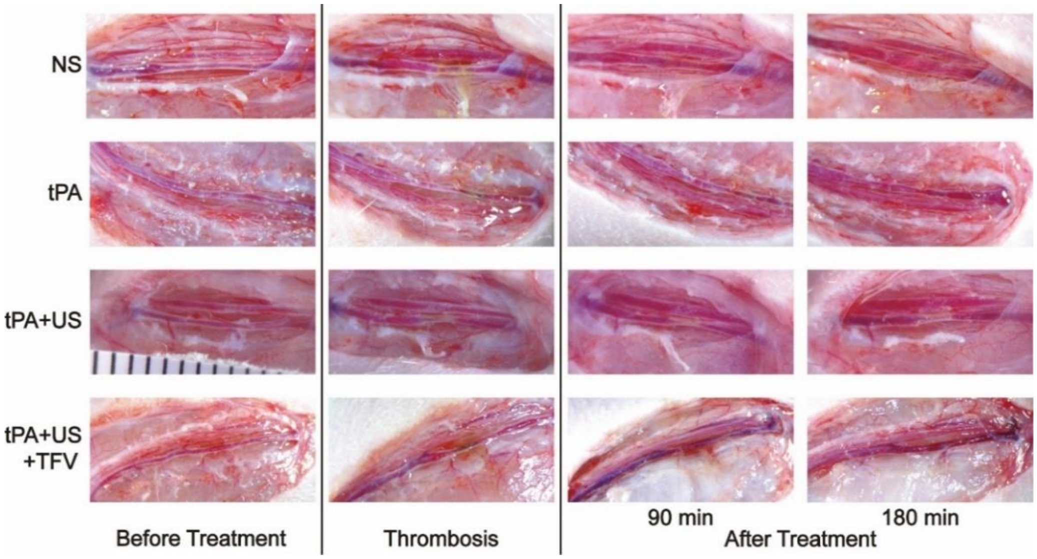

Figure S27. Snapshot of the process captured by the operational microscope under different treatments.

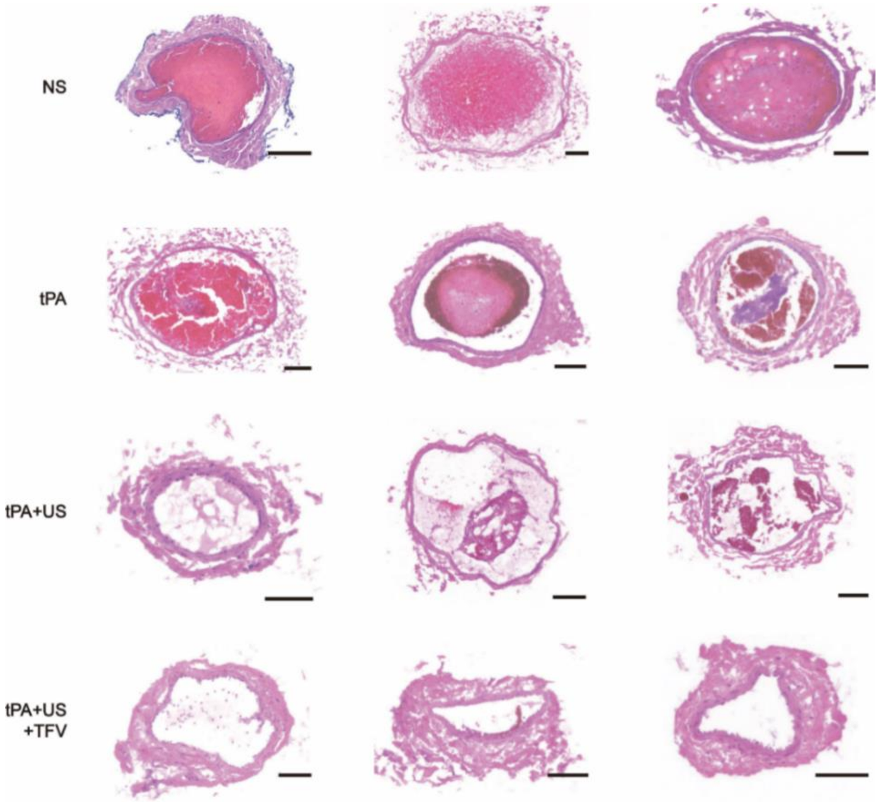

Figure S28. Histological analysis of the femoral vein after treatments with NS, tPA, tPA+US, and tPA+US+TFV for 3 h, respectively (n=3). Scale bar, 100 μm.

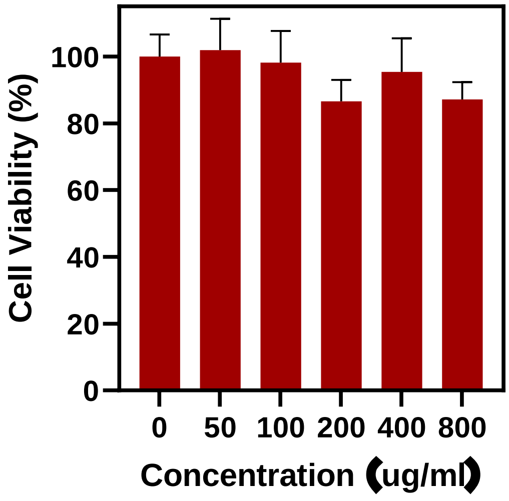

Figure S29. Cytotoxicity of magnetic colloids. The viability of human umbilical vein endothelial cell (HUVEC) was tested by CCK-8 assay after treated with different concentration of magnetic colloidal dispersion solution.

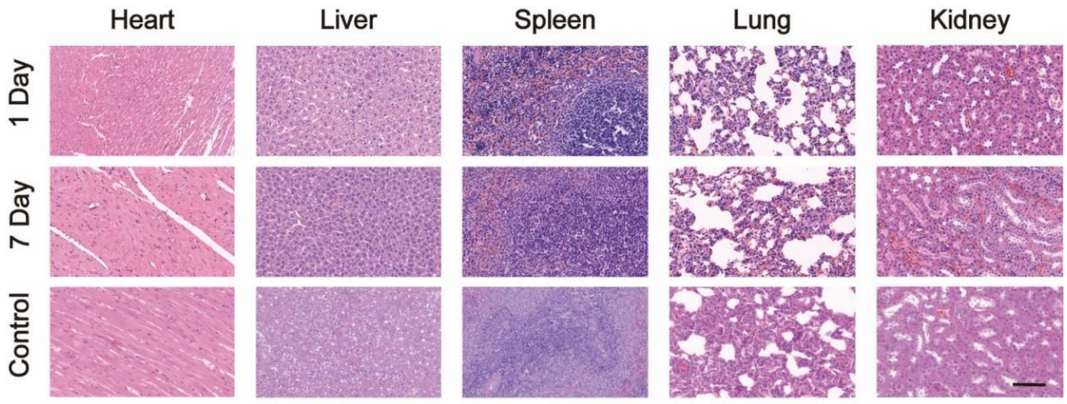

Figure S30. In-vivo safety assessment by H&E staining (n=3 for each group). Scale bar, 100 μm.

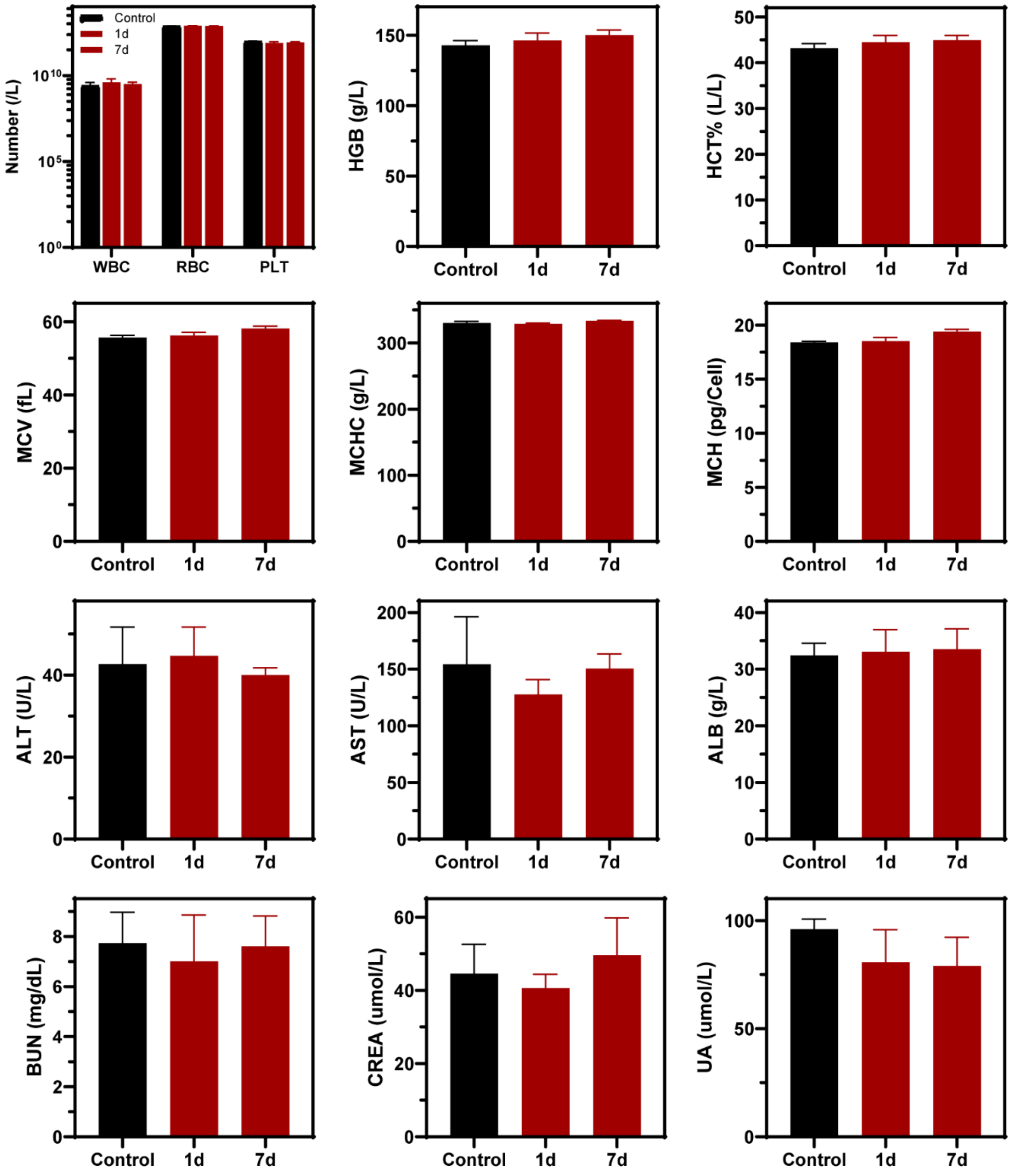

Figure S31. In-vivo safety assessment by blood panel test and serum biochemical analysis.

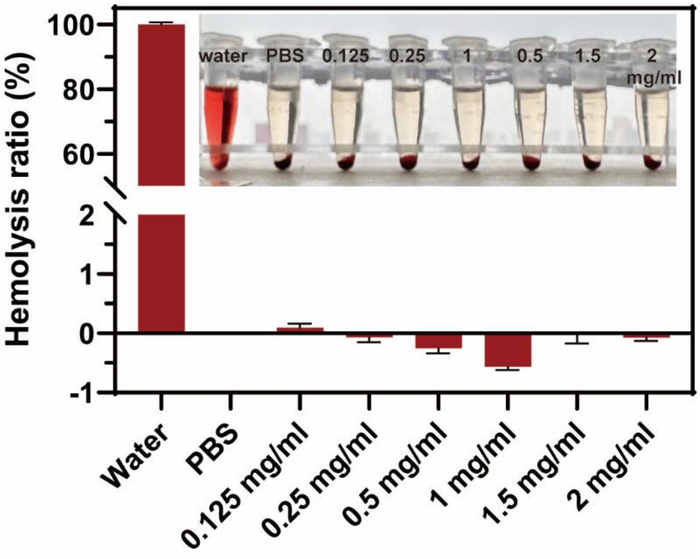

Figure S32. Hemolysis assay of the magnetic colloids toward red blood cells after treated with different concentration of magnetic colloids dispersion solution (0.125, 0.25, 0.5, 1, 1.5, 2 mg/ml).

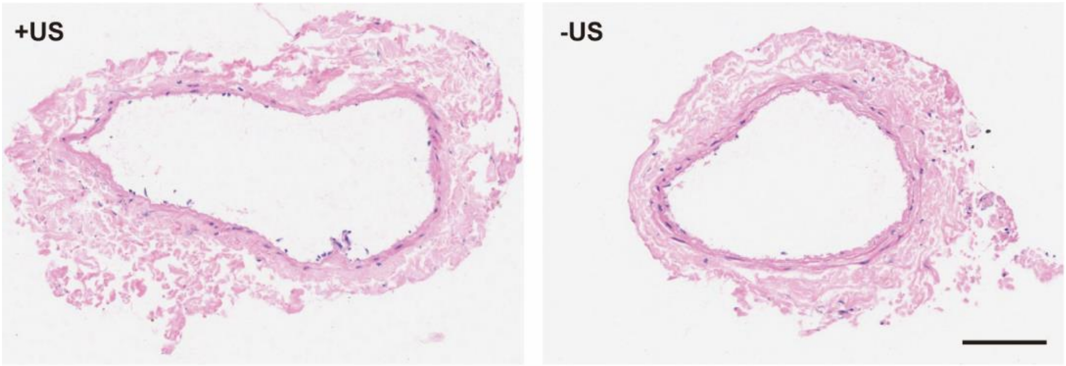

Figure S33. The H&E staining of blood vessels after low-intensity ultrasound irradiation for 3 hours. The result indicated no obvious damage of vascular endothelium was found. Scale bar, 100 μm.
